# A randomised, controlled, two-Centre open-label study in healthy Japanese subjects to evaluate the effect on biomarkers of exposure of switching from a conventional cigarette to a tobacco heating product

**DOI:** 10.1186/s12889-017-4678-9

**Published:** 2017-08-22

**Authors:** Nathan Gale, Mike McEwan, Alison C. Eldridge, Neil Sherwood, Edward Bowen, Simon McDermott, Emma Holmes, Andrew Hedge, Stuart Hossack, Oscar M. Camacho, Graham Errington, John McAughey, James Murphy, Chuan Liu, Christopher J. Proctor, Ian M. Fearon

**Affiliations:** 1 British American Tobacco (Investments) Limited, Research and Development, Regents Park Road, Southampton, SO15 8TL UK; 2 Neil Sherwood Consulting, 22 Route de Marnex, CH-1291 Commugny, Switzerland; 3 Early Clinical Services Medical Writing, Global Medical and Regulatory Writing, Covance Clinical Research Unit Limited, Springfield House, Hyde Street, Leeds, LS2 9LH UK; 4 Early Clinical Development, Covance Clinical and Periapproval Services Limited, Ground Floor, Apsley House, 78 Wellington St, Leeds, LS1 2EQ UK

**Keywords:** Smoking, Biomarker of exposure, Tobacco heating product, Harm reduction

## Abstract

**Background:**

Smoking is a leading cause of numerous human disorders including lung cancer, chronic obstructive pulmonary disease, and atherosclerotic cardiovascular disease. The development of modified risk tobacco products (MRTPs) has been suggested as a possible way to reduce the risks of tobacco smoking by reducing exposure to cigarette smoke toxicants. This study is designed to investigate whether biomarkers of such exposure are reduced when smokers switch from smoking commercial cigarettes to using either a novel or a commercially-available tobacco heating product (THP).

**Design and Methods:**

This study will assess biomarkers of exposure in current smokers who either remain smoking, switch to THP use, or quit all tobacco use completely, for 5 days. The study is an in-clinic (confinement) two-centre, randomised controlled clinical study with a forced-switching design.

Subjects of either gender will be aged 23–55 years (minimum legal smoking age plus 3 years), of Japanese origin and with a verified smoking status (assessed by exhaled breath carbon monoxide and urinary cotinine levels). Subjects will have a usual brand cigarette within the International Organisation for Standardisation (ISO) tar band of 6-8 mg and will be judged to be healthy by medical history, physical examination, vital signs, electrocardiography (ECG), clinical biochemistry and lung function tests.

The primary objective of this study is to assess changes within groups in selected biomarkers of exposure (BoE) and of biological effect (BoBE) after a forced switch from a commercial control cigarette to either a menthol or a non-menthol THP. Secondary objectives are to assess between-group differences, to determine nicotine pharmacokinetics for cigarettes and THPs, to assess subject’s satisfaction with the study products, and to monitor additional endpoints related to safety and product use.

**Discussion:**

Data from this study will advance our scientific understanding of the changes in exposure to cigarette smoke toxicants in smokers who switch to using a THP.

**Trial registrations:**

UMIN000024988 (25th November 2016); ISRCTN14301360 (14th December 2016)

**Electronic supplementary material:**

The online version of this article (doi:10.1186/s12889-017-4678-9) contains supplementary material, which is available to authorized users.

## Background

Smoking is a leading cause of numerous human disorders including lung cancer, chronic obstructive pulmonary disease, and atherosclerotic cardiovascular disease. The increased relative risk of an individual developing a smoking-related disease is associated with both the number of years in which they have been smoking as well as the number of cigarettes smoked daily [5, 13]. The principal factor in this elevated risk is exposure to a number of smoke toxicants which are present in cigarette smoke following the combustion of tobacco in the cigarette [14]. Reducing the deleterious individual and population-level impacts of cigarette smoking on health has become a public health priority, leading to the development of a variety of different initiatives across the world to encourage reducing toxicant exposure through smoking cessation [34]. Despite these efforts, smoking rates in adult populations worldwide remain at 15%–25%. Although numbers are declining slowly in many countries [34], the World Health Organisation (WHO) has forecast that there will be around 1.5 billion tobacco smokers worldwide in 2050 [19]. Current scientific study and public policy debate, therefore, are concerned with whether public health gains could arise from reducing future exposure to toxicants in people who continue to use tobacco through the development of new tobacco products.

Smoke from conventional cigarettes is a complex and dynamic mixture of more than 5600 identified chemical constituents [22], in both its particulate and vapour phases. Some of these chemicals have been identified as potential contributors to the harmful effects of cigarette smoke and can be evaluated by measuring the levels of these chemicals themselves, or their metabolites, in urine. Nicotine, a chemical also found naturally in tobacco leaf and which transfers into cigarette smoke, is primarily responsible for the addictive properties of cigarette smoking. Nicotine is rapidly absorbed into the bloodstream during cigarette smoking [3, 18], from where it is rapidly distributed causing both systemic and central effects. In the central nervous system, nicotine acts at neuronal nicotinic receptors and this interaction may underpin its effects on mood and relaxation [1]. The pharmacokinetic profile of nicotine during cigarette smoking is a rapid rise and fall in plasma nicotine concentrations [7]. Correspondingly, the delivery of nicotine to the brain, and the consequent pleasurable effects experienced by the smoker, are also rapid [1].

In the 2001 report by the US Institute of Medicine (IoM) entitled ‘*Clearing the Smoke: the scientific basis for tobacco harm reduction’* [32], the development of potential reduced-exposure products (PREPs) was suggested as a possible way to achieve tobacco harm reduction. PREPs were defined at that time as products that result in substantial reductions in exposure to one or more tobacco toxicants and that can reasonably be expected to reduce the risk of developing one or more specific diseases or adverse health effects as compared with risks conferred by use of traditional tobacco products. Although the development of a reduced toxicant combustible cigarette with the properties of a PREP has been described, a long-term clinical study failed to show many significant changes in biological indicators of cigarette smoke exposure [30]. Since that time, the development of electronic cigarettes (e-cigarettes) and their rapid evolution has led to a significant body of evidence being developed in support of e-cigarettes as PREPs, evidenced not least by several public health bodies in the UK suggesting that e-cigarette use is likely to be significantly less harmful than cigarette smoking (e.g. [27]). The uptake of e-cigarettes by smokers however has not been universal, suggesting that perhaps in those smokers who would prefer a more cigarette-like experience, other forms of reduced toxicant nicotine delivery systems may be needed to encourage a behavioural change towards a reduced exposure form of tobacco and nicotine use.

Tobacco heating products (THPs) are electronic devices that heat a specially designed tobacco stick, typically to temperatures lower than 350 °C. It is important to distinguish THPs from e-cigarettes, which heat a tobacco-free nicotine solution. This temperatures at which THPs operate do not facilitate tobacco combustion and as a consequence far fewer chemical toxicants are formed and of those formed, the majority are reduced significantly. However, data from this and other similar tobacco products suggests that nicotine is still released into the inhaled aerosol to a level not dissimilar to that of conventional cigarettes [23]. THP sticks are similar in appearance to a conventional cigarette and include a filter section at the mouth end of the tobacco stick and contain processed tobacco wrapped by paper. Less is known concerning the properties of THPs compared to conventional cigarettes when actually consumed by humans, both in terms of nicotine delivery and toxicant exposure. In studies in subjects who have switched from smoking to using a commercially available electrically-heated THP, reductions in exposure to a wide range of smoke chemicals have been reported. These effects were rapidly attained and sustained for a period of at least one month of continued use [9, 12, 17, 20, 28, 33]. In this study we propose to examine exposure to smoke toxicants, using biomarkers of exposure, as well as nicotine pharmacokinetics, in smokers who use a newly-developed THP.

### Study objectives

The primary aim of this study is to examine biomarkers of exposure (BoE; Table 1) to cigarette smoke toxicants in the breath and urine of smokers who either remain smoking combustible cigarettes, or switch to using a THP device, for 5 days. This will be benchmarked against the reductions seen when a cohort of smokers abstain from the use of any tobacco product for the 5-day period. We further aim to investigate the effect of switching from a cigarette to using a THP on 2 biomarkers of biological effect (BoBE), to determine the pharmacokinetics of nicotine delivery in subjects smoking a conventional cigarette or using a THP, and to examine additional endpoints related to safety, product use, satisfaction and the nicotine metabolite ratio (Table 1).

**Table 1:** Study endpoints

BoE	Abbreviation	Associated Toxicant	Matrix
Carbon monoxide	CO	Carbon monoxide	Exhaled breath
Total nicotine equivalents (nicotine, cotinine, 3-hydroxycotinine and their glucuronide conjugates)	TNeq	Nicotine	Urine
Total 4-(methylnitrosamino)-1-(3-pyridyl)-1-butanol	Total NNAL	4 (methylnitrosamino)-1-(3-pyridyl)-1-butanone (NNK)	Urine
Total N-nitrosonornicotine	Total NNN	NNN	Urine
3-hydroxypropylmercapturic acid	3-HPMA	Acrolein	Urine
3-hydroxy-1-methylpropylmercapturic acid	HMPMA	Crotonaldehyde	Urine
S-phenylmercapturic acid	S-PMA	Benzene	Urine
Monohydroxybutenyl-mercapturic acid	MHBMA	1,3-butadiene	Urine
2-cyanoethylmercapturic acid	CEMA	Acrylonitrile	Urine
4-aminobiphenyl	4-ABP	4-Aminobiphenyl	Urine
o-toluidine	o-Tol	o-Toluidine	Urine
2-aminonaphthalene	2-AN	2-Aminonaphthalene	Urine
1-hydroxypyrene	1-OHP	Pyrene	Urine
2-hydroxyethylmercapturic acid	HEMA	Ethylene oxide	Urine
N-acetyl-S-(2-carbamoylethyl)cysteine and N-acetyl-S-(2-hydroxy-2-carbamoylethyl)cysteine	AAMA and GAMA	Acrylamide	Urine
BoBE	Abbreviation	Biological Effect	Matrix
8-Epi-Prostaglandin F2α Type III	8-Epi-PGF2_α_ Type III	Oxidative stress	Urine
White blood cell count	WBC count	Inflammation	Whole blood
Nicotine pharmacokinetic parameters		Abbreviation	
Time to maximum observed plasma concentration for nicotine		t_max_	
Maximum observed plasma concentration for nicotine		C_max_	
Area under the nicotine plasma concentration-time curve from time zero to the last measurable concentration		AUC_0-last_	
Safety parameters		Other measures	
Medical history of study subjects		Product satisfaction questionnaire	
Physical examination		Puff count during pharmacokinetic assessment period	
Vital signs		Creatinine concentration (24-h urine)	
Electrocardiogram (ECG)		Product use during exposure phase	
Clinical laboratory evaluations		Nicotine molar metabolite ratio	
Lung function test			
Adverse event /serious adverse event recording			

*BoE* biomarker of exposure, *BoBE* biomarker of biological effect

## Methods/design

### Study registrations

This study was registered on the ISRCTN registry on 14th December 2016 and given the registration number ISRCTN14301360. The study was also registered on the UMIN registry on 25th November 2016 and given the registration number UMIN000024988.

### Study design

This study will be a randomised, controlled clinical trial with a forced-switching design that will be conducted in 2 clinics in Fukuoka, Japan. This study will be conducted in compliance with the ethical principles of the Declaration of Helsinki, Good Clinical Practice (International Conference on Harmonisation (ICH) E6 Consolidated Guidance, April 1996) and Japanese laws, including those relating to the protection of subjects’ personal data. All subject names and other potential identifiers will be filed confidentially by the Principal Investigator (PI). Subjects will be identified in documentation and throughout evaluation by the number allotted to them during the study (see section on Randomisation).

The PI will ensure that participants are given full and adequate oral and written information in non-technical terms about the nature, purpose, potential risks and any possible benefits of study participation. Participants will be given time to consider all the information, the opportunity to ask questions and will be required to read, sign and date the informed consent form (ICF; Additional file 1: Appendix 1) that summarises the discussion before participating in any procedures related to the study. The protocol (Version 2.0 dated 17th November 2016) and the ICFs (Additional file 1: Appendix 1) were submitted for review to the Hakata Clinic Institutional Review Board (IRB), Medical Co. LTA, Fukuoka, Japan**.** A favourable opinion from the IRB was obtained on 16th December 2016 (reference number 1684CP). All substantial protocol amendments will be approved by the IRB responsible for the study and the IRB will be informed of minor amendments to the protocol which do not require their approval.

The study has a forced-switching design to enable data to be obtained about changes in exposure to tobacco smoke toxicants and related biological effects. The effects of THP use will thus be evaluated within individuals (switching smokers serve as their own control) and between individuals (switching versus non-switching smokers). A cessation group will provide information on changes in biomarkers in subjects who quit smoking or use of any tobacco products. The cigarette and cessation comparators were chosen to enable comparison of toxicant exposure in THP users with those of combustible cigarette smokers and smokers who have completely refrained from smoking, respectively.

Financial compensation for the inconvenience to and effort of participants will be offered as part of the study, but the Sponsor does not wish this approach to incentivise participants to smoke. Stipends will, therefore, be calculated independently by the clinics according to the usual rates for this type of clinical study and will be approved by the Hakata Clinic IRB. The stipend therefore offers fair compensation for the inconvenience and effort required of the subjects. Other than subjects in the cessation group, who will receive slightly increased compensation due to the additional discomfort of ceasing all nicotine use for five days, all subjects will receive the same amount of compensation on completion of the study.

Inclusion and exclusion criteria have been carefully tailored so as not to recruit any smoker who is planning to quit smoking in the next 12 months. This study does not force smokers to smoke. The ICF to be signed by each subject will specify the requirements of the study and the risks associated with participation in the study. During the exposure phase of the study there are no specified smoking times, the smoker chooses if and when they smoke. At in-clinic evaluations the subjects must request a cigarette/THP when they wish to smoke. A maximum daily limit of product use will be specified, based on 120% of subjects’ usual cigarette consumption, to prevent subjects from smoking to a degree greater than they would normally. Such an uplift in smoking behaviour has been observed during clinical studies with cigarettes [31]. During the pharmacokinetic phase, subjects will be requested to smoke 1 cigarette/THP at a specified time.

The age of each subject will be verified using appropriate identification documentation prior to inclusion in the study to ensure subject age restrictions specified in this protocol are complied with.

### Study participants

#### Selection of study population

In total, 180 male or female smokers will be enrolled in this study. Smokers whose current brand of cigarettes is mentholated or non-mentholated will be enrolled, with the former being randomised only into the menthol product use or cessation groups, and the latter only into the non-menthol product use or cessation groups. Subjects of each gender will have a quota applied to ensure that they represent at least 40% of the total population and/or of each group. Ongoing monitoring of recruitment logs by the clinical sites will be performed to ensure that the gender split in the groups matches the protocol requirements.

#### Identification of study participants

Participants will be recruited into the study through advertisements on the clinic’s own website and local advertising that will not refer to the characteristics of the study products. Adult, healthy participants of either sex and of Japanese origin will be enrolled.

#### Inclusion criteria

The suitability of participants who give informed consent will be assessed according to the inclusion criteria within 35 days before entering the study and verified upon arrival at the clinic for the in-clinic evaluation period. The universal inclusion criteria are current smokers (as verified at screening and admission using a urinary cotinine test (cotinine >200 ng/mL) and exhaled breath carbon monoxide (CO) test (CO >10 ppm)) who report typically smoking at least 10 and a maximum of 30 commercially available cigarettes per day, within the ISO tar bands of 6 mg to 8 mg; at least 3 years’ consecutive smoking history including smoking their preferred brand of cigarettes for at least 6 months immediately prior to screening; age between 23 and 55 years inclusive; weight of at least 50 kg (men) or 40 kg (women) and body mass index (BMI) between 17.6 and 32.0 kg/m^2^, inclusive; no clinically-relevant abnormal findings on physical examination, vital signs assessment, ECG, clinical laboratory testing or lung function tests, or in the medical history, as judged by the investigators; the ability to communicate well with the investigators and understand and comply with the requirements of the study; willingness to refrain from consuming alcohol within 72 h before screening and the in-clinic evaluation; willingness to refrain from consuming and avoid being in the presence of the cooking of cruciferous vegetables and grilled, fried or barbequed food for 48 h before the first day of the in-clinic evaluation; willingness to avoid eating foods containing poppy seeds for 72 h prior to screening and the in-clinic evaluation; if of non-childbearing potential provide confirmation; if of childbearing potential subjects must not be pregnant or breastfeeding and must be using an acceptable method of contraception from from the time of signing the ICF until the end of the safety follow-up period; or being postmenopausal with amenorrhea for at least 1 year.

Additional inclusion criteria are; willing to use the study products (comparator cigarette or THP) and smoke only the products provided to them during clinical confinement, or to abstain from smoking if assigned to the cessation group.

#### Exclusion criteria

Exclusion criteria may be applied at screening or at any time during the study. The universal exclusion criteria are male subjects who do not agree, or whose partners of childbearing potential do not agree, to use a barrier method of contraception (i.e., a condom with spermicide) in addition to a second highly effective method of contraception used by their female partners or to refrain from donating sperm from admission (Day −1) until the end of the safety follow-up period (5–7 days after discharge); female subjects of childbearing potential who do not agree to use a highly effective method of birth control in conjunction with male barrier method contraception (i.e. a condom with spermicide) from the time of signing the ICF until the end of the safety follow-up period (5–7 days after discharge); female subjects who are pregnant or breastfeeding (this will be confirmed at screening and admission and any female subject who becomes pregnant during this study will be withdrawn); subjects who have donated ≥400 mL of blood within 12 weeks (males) or 16 weeks (females) prior to admission, plasma in the 2 weeks prior to admission or platelets in the 6 weeks prior to admission; subjects who have had an acute illness (e.g. upper respiratory tract infection, viral infection, etc.) requiring treatment within 4 weeks prior to admission; subjects who have regularly used any nicotine or tobacco product other than factory-made filter cigarettes within 14 days of screening; subjects who are self-reported or observed at admission by the clinic staff as non-inhalers (smokers who draw smoke from the cigarette into the mouth and throat but who do not inhale); subjects who have a significant history of alcoholism or drug/chemical abuse within 24 months prior to screening, as determined by the PI; subjects who have a positive urine drugs of abuse screen or alcohol breath test (either confirmed by repeat) at screening or admission; subjects who have serum hepatitis, are carriers of the hepatitis B surface antigen (HBsAg), are carriers of the hepatitis C antibody or have a positive result for the test for human immunodeficiency virus (HIV) antibodies, or have syphilis; subjects who have used prescription or over-the-counter (OTC) bronchodilator medication (e.g. inhaled or oral β-adrenergic agonists) to treat a chronic condition within the 12 months prior to admission; subjects who have received any medications or substances (other than tobacco) which interfere with the cyclooxygenase pathway (e.g. anti-inflammatory drugs including aspirin and ibuprofen) within 14 days prior to admission or which are known to be strong inducers or inhibitors of cytochrome P450 (CYP) enzymes within 14 days or 5 half-lives of the drug (whichever is longer) prior to admission; subjects who perform strenuous physical activity (exceeding the subject’s normal activity levels) within 7 days prior to screening or admission; subjects who are unable to communicate effectively with the PI/study staff (i.e. language problem, poor mental development, or impaired cerebral function); subjects who are unwilling or unable to comply with the study requirements; employees and immediate relatives of those employed in the tobacco industry or the clinical site; subjects who are still participating in another clinical study (e.g. attending follow-up visits) or who have participated in a clinical study involving administration of an investigational drug (new chemical entity) in the past 3 months prior to first product use; subjects who have any clinically relevant abnormal findings on the physical examination, medical history, ECG, lung function tests (forced expiratory volume after 1 s [FEV_1_]/forced vital capacity [FVC] <0.7 at post-bronchodilator spirometry, post-bronchodilator FEV_1_ < 80% predicted value, and post-bronchodilator FVC <80% predicted value) or clinical laboratory panel, unless deemed not clinically significant by the PI or their appropriately qualified designee; subjects who have, or who have a history of, any clinically significant neurological, gastrointestinal, renal, hepatic, cardiovascular, psychiatric, respiratory, metabolic, endocrine, haematological or other major disorder that, in the opinion of the PI or their appropriately qualified designee, would jeopardise the safety of the subject or impact on the validity of the study results; subjects who have previously been diagnosed with any form of malignancy; subjects who have any clinically significant abnormal laboratory safety findings at screening and prior to first product use, as determined by the PI (1 repeat assessment is acceptable); subjects who have previously taken part in or withdrawn from this study; and subjects who, in the opinion of the PI, should not participate in this study.

One further exclusion criterion is that subjects will be excluded from study participation if, prior to enrolment, they are planning to quit smoking in the next 12 months. All subjects will be informed that they are free to quit smoking and withdraw from the study at any time. Any subject who decides to quit smoking will be directed to appropriate stop smoking services.

#### Withdrawal from the study

All subjects will be reminded of the harms associated with smoking prior to enrolment onto the study and that they are free to voluntarily quit smoking and/or withdraw from the study at any time. If they do so, they will receive a pro-rata stipend. Investigators may also at any time withdraw a participant from the study if they deem this action to be in his or her best interest or that of other participants. The end-of-study examination will be undertaken when a participant leaves the study, if he or she agrees, and reasons for withdrawal will be recorded. If a subject cannot be assessed because he or she cannot be contacted after non-attendance of a scheduled event, they will be reported as lost to follow-up.

Subjects may be withdrawn from the study prematurely for the following reasons: if a subject experiences an intolerable adverse event (premature discontinuation will be at the discretion of either the PI or the subject, independent of the relationship of the AE to the study test product); if a subject develops a non-fulfilment of inclusion/exclusion criteria or concurrent disease, which at the discretion of the PI, no longer justifies the subject’s participation in this study; or if any deviations occur during the conduct of the study- this may include the subject’s erroneous inclusion in the study. Any protocol deviations detected during the study will be corrected when possible and the subject will be allowed to continue. All protocol deviations will be fully documented and considered for their effect on study objectives. Deviations that could lead to subject discontinuation from the study include: deviations which could affect subject’s safety (e.g. illness requiring treatment[s]) which in the clinical judgement of the PI (or after discussion with the Medical Monitor) might invalidate the study by interfering with the allocated test product or the willingness of the subject to comply with the study activities; deviations involving the use of any nicotine/tobacco products other than the intended conventional cigarettes or THPs; or deviations involving the use of any nicotine/tobacco products by the cessation group during the exposure period. A subject may also be withdrawn from the study: if the subject is uncooperative, including non-attendance (in these cases, efforts should be made by the PI to ascertain the reason and to ensure subject’s attendance as soon as possible); due to pregnancy; due to premature cancellation of the study; or at the subject’s personal request: the subject could decide, at any moment of the study, to stop his/her participation. The PI should ensure this is not due to AEs, in which case, this reason should be documented. Subjects do not have to provide a reason for withdrawing from the study if they do not wish to do so. Although withdrawals or dropouts are not expected to be replaced, the Sponsor will consider replacing participants to maintain the power of the study.

### Smoking advice and support

Information will be provided regarding the health risks associated with smoking. Advice on smoking cessation will be freely available to all subjects at screening, admission, and discharge. The advice will be based on the recommendations of the WHO “Evidence based Recommendations on the Treatment of Tobacco Dependence”. Subjects who decide to quit smoking during the study period will be referred to the appropriate stop smoking services to support their cessation attempt.

### Investigational products

All study cigarettes and THP devices/tobacco sticks will be provided by the Sponsor free of charge. Brief product details and codes are shown in Table 2. A single study product will be allocated to each group; these groups are (A) non-mentholated combustible tobacco cigarette; (B) glo device with non-mentholated Neostik; (C) mentholated combustible tobacco cigarette; (D) glo device with mentholated Neostik and (E) iQos device with Marlboro non-mentholated Heatsticks. A further (abstinence) group (F) will refrain from using any tobacco or nicotine products after the switch at the end of the baseline period.

**Table 2:** Investigational Products. Details of the investigational products to be used in this study. The target values for nicotine content are manufacturing specifications and the actual results may vary due to product variation, but these values will be within an appropriate range

Study Product Reference	Format	Product & Manufacturer	ISO Nicotine Yield	ISO Tar Yield
A	Combustible tobacco cigarette (non-menthol)	Lucky Strike Regular, BAT	0.63 mg/cig	7 mg/cig
B	THP (non-menthol)	Glo device with Neostik, BAT	0.47 mg/stick	N/A
C	Combustible tobacco cigarette (menthol)	Lucky Strike Menthol, BAT	0.63 mg/cig	7 mg/cig
D	THP (menthol)	Glo device with mentholated Neostik, BAT	0.47 mg/stick	N/A
E	THP (non-menthol)	iQos device with Marlboro Heatsticks^#^	0.4 mg/stick	N/A

*BAT* British American Tobacco, *THP* tobacco heating product, *THD* tobacco heating device, *ISO* International Organisation for Standardisation. ^#^Commercially-available products manufactured by Philip Morris International and procured from general a retail source in Japan

The combustible cigarettes (Products A and C) and Product E are commercially available products

Although this study is unblinded due to the obvious differences between cigarettes and THP tobacco sticks, unbranded conventional cigarettes and packs will be manufactured by BAT specifically for the study. The commercially-available THP and tobacco sticks will be acquired directly from the market. All packaging will include the mandatory health warnings. The different tobacco products will be identifiable to the clinic pharmacy staff and investigators by study identification codes forming part of study-specific labels applied to the product packaging.. The tobacco products will be stored in a locked, limited-access area under controlled temperature conditions.

**Table 3:** Schedule for baseline study period (Days −1 to 2)

Day	Start of Procedure	Procedure
-1	12:00–19:00	Admission procedures: medical history, supine BP, PR, RR, axillary body temperature, abbreviated physical examination, lung function test, drug screen, alcohol breath test, urine cotinine test, exhaled CO test, urine pregnancy test for females, product test, dinner
	19:00–20:00	Start of urine collection, start of monitoring conventional cigarette consumption
	20:00–22:00	Snack
	00:00	Designated smoking room closed
1	06:00	Designated smoking room opened for use
	06:00–09:00	Breakfast
	12:00–14:00	Lunch
	16:30–17:30^a^	Exhaled CO assessment
	18:00–19:00	Dinner
	19:00–20:00^b^	End of first 24-h urine and cigarette use monitoring, start of second 24-h urine and cigarette use monitoring
	20:00–22:00	Snack
	00:00	Designated smoking room closed
2	06:00	Designated smoking room opened for use
	06:00–09:00	Breakfast
	12:00–14:00	Lunch
	15:30–16:30^c^	Blood sample collection for WBC
	16:30–17:30^a^	Exhaled CO assessment
	18:00–19:00	Dinner

*BP* blood pressure, *CO* carbon monoxide, *PR* pulse rate *RR* respiration rate, *WBC* white blood cells. ^a^Exhaled CO assessments for individual subjects should be taken at the same time on each sampling day. ^b^End of 24-h urine collection needs to be time-matched to the start of 24-h urine collection for each individual subject. ^c^Time of blood sampling for WBC for individual subjects should be taken at the same time on each sampling day

#### Randomisation

Separate randomisations will be provided for each site and each site will need to recruit 15 subjects for each arm. In total 3 randomisation lists will be produced. Arms A and B will be studied first so the first 60 subjects will be randomised to these two arms. Male subjects will be assigned starting at subject number 0001 onwards and female subjects will be assigned starting at subject number 0060 downwards. The remaining 2 lists will be produced for arms E and F (non-menthol users) and for arms C and D (menthol users). In each list 60 subjects will be randomised so that 30 subjects are assigned to each arm. Male subjects will be assigned starting at subject numbers 0061 and 0121 onwards in each list respectively and female subjects will be assigned starting at subject numbers 0120 and 0180 downwards in each list respectively. A suitably qualified person will monitor the randomisation to ensure there is at least 40% of each gender in an arm.

The randomisation will be performed by Covance and the clinics will enrol the participants and assign them to interventions. A suitably qualified person will monitor the randomisation to ensure there is at least 40% of each gender in an arm.

#### Compliance

The study will be conducted under clinical confinement and subjects will not be allowed to bring their own cigarettes/THPs into the clinic. To ensure product use compliance in the clinic, all products will be used by subjects under the supervision of suitably qualified staff. Study products will be dispensed by clinic staff each time a study subject wishes to smoke a cigarette or use a THP. Records will be kept of all product usage and these records will be checked by the Clinical Research Associates (CRAs). Subjects in the cessation arm will neither be provided with cigarettes/THPs nor be allowed to enter the smoking rooms. Subjects randomised to one of the THP groups will receive training by clinic staff on how to operate their assigned THP device. The THPs will be charged and assembled for the subjects, who will only be required to activate the devices. Product information sheets will be provided for each of the THP test products.

### Concomitant medication

Subjects should avoid medication that interferes with the cyclooxygenase pathway (anti-inflammatory drugs such as aspirin and ibuprofen) within 14 days prior to admission until discharge at the end of the study (end of Day 7 for Group F or end of Day 8 for Groups A to E). Subjects should also not use any drugs or substances (except tobacco) known to be strong inducers or inhibitors of CYP enzymes (formerly known as cytochrome P450 enzymes) within 14 days or 5 half-lives of the drug (whichever is longer) prior to admission (Day −1).

No prescription or OTC medications (including herbal medications but excepting hormonal contraceptives) should be taken during the course of the study. However, medications considered necessary for the welfare of the subject, and which are not expected to interfere with the evaluation of the investigational products may be permitted at the discretion of the PI, for example, occasional use of paracetamol in case of headache of up to 4 g/day.

Should the need arise to take any medications the PI should be informed as soon as possible, if safety allows this should be reported beforehand. If any medication is required, the name, strength, frequency of dosing and reason for its use will be documented in the subject’s electronic case record form (eCRF) by the PI or their appropriately qualified designee.

### Diet

During the in-clinic evaluation at the study site, subjects will receive a standardised bulk diet at scheduled times which do not conflict with other study-related activities. In order to minimise interfering substances, the diet provided will exclude cruciferous vegetables and grilled, smoked, fried or barbequed food items. These dietary restrictions will be similar for all study groups. Consumption of water will be freely permitted during the study.

### Study procedures

**Fig. 1:**
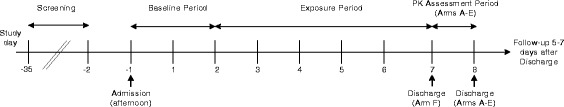
Outline of study procedures

An outline of the study procedures is shown in Fig. 1 and described in detail below. In brief, following screening, subjects who meet all inclusion/exclusion criteria will be entered onto the study. On Day −1, subjects will enter the clinic and 24 h urine collection periods will begin, for 2 days. After this period and according to the randomisation code, subjects will either remain smoking regular cigarettes, switch to using a THP, or refrain from using any tobacco products, for a period of 5 days. During this period we will continue to collect 24 h urine samples for BoE analysis. At the end of this 5 day period those in the cessation group will be discharged from the clinic following safety assessments. Subjects in the smoking and THP groups will remain in the clinic for a further day, in order for us to determine their nicotine pharmacokinetic parameters during a single use of their allocated tobacco product. This will be obtained following at least a 12-h abstention from the use of any tobacco products. Following blood sampling for pharmacokinetic analysis subjects will be discharged from the clinic following safety assessments. All subjects will further be followed up by telephone 5–7 days after their last in-clinic day, to capture any adverse events.

#### Telephone screening

Subjects will undergo telephone screening within 34 days prior to the first product use. Staff will verbally confirm that the subjects comply with the inclusion/exclusion criteria prior to attending the site for a screening visit.

#### Screening

Following the telephone screening and prior to first product use, subjects will attend a screening visit at one of the clinical sites. Prior to the screening visit, subjects will refrain from strenuous physical activity (exceeding the subject’s normal activity levels) for 7 days and abstain from alcohol for 72 h. Subjects will be asked to sign the study-specific ICF in the presence of a suitably trained clinical site clinical operations staff member prior to any screening procedures being performed. The information recorded for all subjects, regardless of their suitability for the study, will be retained and archived in accordance with the PI’s data retention policies.

Subjects will be asked to provide a urine sample for a cotinine screen to ensure that they are currently using nicotine-delivering products. Subjects will also be asked to provide an exhaled breath sample for a CO test to ensure that they smoke combustible cigarettes. They will only be enrolled into the study if their urine cotinine level is greater than 200 ng/mL and their exhaled breath CO level is greater than 10 ppm.

The following information and procedures will also be recorded and performed as part of the screening assessments: medical history; gender, race/ethnic origin, age, height, weight and BMI; vital signs, including supine blood pressure, supine pulse rate, respiratory rate, and axillary body temperature; resting 12-lead ECG; physical examination (may be performed at admission); lung function tests; urine drugs of abuse screen and alcohol breath test; clinical laboratory evaluations; hormone panel analysis (female subjects only); and nicotine use assessment (including tobacco and nicotine use history and Fagerström test for cigarette dependence (FTCD) questionnaire). All subjects will be given a demonstration of the THP products at screening.

#### Restrictions prior to clinical study visit

Subjects will be advised to refrain from consuming cruciferous vegetables, and grilled, smoked, fried or barbequed food for 48 h prior to admission. This will also include being in the presence of the cooking of cruciferous vegetables, and grilled, smoked, fried or barbequed food. Subjects will be asked to confirm this as part of the clinic admission procedure on Day −1.

Subjects will be advised that they must not eat food containing poppy seeds for 3 days before both screening and admission (Day −1), as consumption of poppy seeds can lead to a positive opiate result in the drugs of abuse test.

Subjects will be instructed to refrain from consuming alcohol for 72 h prior to admission (Day −1) and will be requested not to undertake vigorous exercise outside of their usual routine from 7 days before Admission (Day −1) until after the post-study assessments.

#### Contraception

Female subjects participating in the study who are of non-childbearing potential will not be required to use contraception. Women of non-childbearing potential are defined as permanently sterile (i.e. due to hysterectomy, bilateral salpingectomy, bilateral oophorectomy, or confirmed tubal occlusion) or postmenopausal (defined as at least 12 months post-cessation of menses without an alternative medical cause). Postmenopausal status will be confirmed with a screening serum follicle-stimulating hormone (FSH) level greater than 40 mIU/mL.

Female subjects of childbearing potential will be required to follow contraception requirements from the time of signing the ICF until the end of the safety follow-up period (5–7 days after discharge). Male subjects and their partners of childbearing potential will be required to follow contraception requirements from admission (Day −1) until the end of the safety follow-up period (5–7 days after discharge).

Women of childbearing potential should be using one of the following acceptable methods of contraception: combined (oestrogen and progestogen containing) oral, intravaginal or transdermal hormonal contraception associated with inhibition of ovulation; progestogen-only hormonal contraception, either oral, injected or implanted, associated with inhibition of ovulation; progestogen-only oral hormonal contraception where inhibition of ovulation is not the primary mode of action; intrauterine device (IUD); intrauterine hormone-releasing system (IUS); bilateral tubal occlusion; male or female condom with spermicide; cap, diaphragm or sponge with spermicide.

Subjects who practice true abstinence, which must be due to the subject’s lifestyle choice; i.e. the subject should not become abstinent just for the purpose of study participation, are exempt from contraceptive requirements. Periodic abstinence (e.g. calendar, ovulation, symptothermal, post-ovulation methods) and withdrawal are not acceptable methods of contraception.

For male subjects, sexual intercourse with female partners who are pregnant or breastfeeding should be avoided unless condoms are used, from admission (Day −1) until the end of the safety follow-up period (5–7 days after discharge). Male subjects are required to refrain from donation of sperm from admission (Day −1) until the end of the safety follow-up period (5–7 days after discharge).

For subjects who are exclusively in same sex relationships, contraceptive requirements will not apply.

#### In-clinic evaluations

From admission until discharge, the only permitted nicotine intake will be from subjects’ assigned study products (cigarette or THP). Subjects randomised to the cessation group (Group F) will be instructed to abstain from all product use after the baseline period until they are discharged at the end of Day 7. Subjects for the pharmacokinetic assessment (Groups A to E) will be instructed to abstain from use of any nicotine-containing products for a minimum of 12 h prior to the pharmacokinetic assessment after which a single product use session will occur.

#### Baseline period

The baseline period will begin at admission on the afternoon of Day −1 and will end on the evening of Day 2 (Table 3). At admission, it will be confirmed that the subject continues to meet all of the study inclusion/exclusion criteria. Any subject who is eligible at screening, but who fails to meet the criteria at admission will be considered a screening failure and will be replaced. Subjects who do not fulfil the entry criteria prior to enrolment, but who are considered eligible, will be immediately withdrawn from the study when the non-fulfilment is detected. If the subject has not yet been enrolled into the study, they can be replaced.

Any changes in medical history since last visit will be discussed with the subject (including intake of any excluded medications) and documented. Subjects will be required to give any tobacco or nicotine products that they have with them to the clinic staff upon admission (these will be returned at the end of the study).

Subjects will be excluded from the study if they have used any nicotine or tobacco product other than commercially manufactured filter cigarettes within 14 days of screening. Only subjects willing to smoke only the cigarettes or THP distributed by the clinical site staff during the in-clinic evaluation period will be enrolled into the study. Subjects will be instructed on how to request their assigned product and return the extinguished cigarette/THP.

#### Exposure period

The exposure period will begin on the evening of day 2 and will continue until the evening of day 7 (Table 4). The subjects will be switched to their assigned product (conventional cigarette, assigned THP or cessation) on the evening of day 2 after completion of the 24-h urine collection. All subjects undergo the same procedures with the exception of the cessation group, Group F, who will not be allowed to use any products during the exposure period.

**Table 4:** Schedule for exposure periods (Day 2 evening – Day7)

Day	Start of Procedure	Procedure
2	19:00–20:00 ^c^	End of second 24-h urine and product use monitoring, start of third 24-h urine and product use monitoring. Switch to next assigned product (Groups A to E) or cessation (Group F)
	20:00–22:00	Snack
	00:00	Designated smoking room closed
3, 4, 5, 6	06:00	Designated smoking room opened for use
	06:00–09:00	Breakfast
	12:00–14:00	Lunch
	15:30–16:30^a^	Blood sample collection for WBC (Day 5 only)
	16:30–17:30^b^	Exhaled CO assessment
	18:00–19:00	Dinner
	19:00–20:00^c^	End of 24-h urine and product use monitoring, start of next 24-h urine and product use monitoring
	20:00–22:00	Snack
	00:00	Designated smoking room closed
7	06:00	Designated smoking room opened for use
	06:00–09:00	Breakfast
	12:00–14:00	Lunch
	15:30–16:30^a^	Blood sample collection for WBC
	16:30–17:30^b^	Exhaled CO assessment
	18:00–19:00	Dinner
	19:00–20:00^c^	End of 24-h urine and product use monitoring End of product use Groups A to E

*CO* carbon monoxide, *WBC* white blood cells. ^a^Time of blood sampling for WBC for individual subjects should be taken at the same time on each sampling day. ^b^Exhaled CO assessments for individual subjects should be taken at the same time on each sampling day. ^c^End of 24-h urine collection needs to be time-matched to the start of 24-h urine collection for each individual subject

#### Nicotine pharmacokinetic assessment period

The nicotine pharmacokinetic assessment period will be from collection of the final 24-h urine sample on Day 7 until discharge on the afternoon of Day 8 (Table 5). All subjects in Groups A to E will remain in the clinic for the pharmacokinetic assessment period. These subjects will undertake a minimum 12-h period of smoking abstinence before being asked to use the cigarette or THP assigned to them during the exposure phase in a single-use session. Subjects will not consume food for a minimum of 1 h before the start of the pharmacokinetic assessment period until the end of the pharmacokinetic assessment period.

**Table 5:** Schedule for nicotine pharmacokinetic assessment

Day	Start of Procedure	Procedure
7	19:00–20:00^a^	Start of product use abstinence
	20:00–22:00	Snack
8	06:00–08:00^b^	Light breakfast / snack
	08:00–11:00	Start of pharmacokinetic assessment period
		• Pharmacokinetic blood draws
		• Product satisfaction questionnaire
	12:00–15:00	End of pharmacokinetic assessment period

^a^Following completion of final 24-h urine collection period. ^b^Light breakfast / snack will be completed at least 1 h prior to the start of the pharmacokinetic assessment period

#### Discharge

Subjects will be discharged from the clinical site following their final 24-h urine collection on Day 7 (Group F) or after their last pharmacokinetic assessment on Day 8 (Groups A-E). The PI will check on all subjects’ wellbeing prior to discharge and provide cessation advice. If necessary, subjects will remain at the clinical site until any AEs of concern have resolved. Discharge assessments will include adverse events, vital signs, physical examination (abbreviated), ECG, lung function test, clinical laboratory assessments and pregnancy test (women of childbearing potential only). Subjects in all groups, should they wish to continue smoking after the study, will be advised to smoke their first post-study cigarette prior to leaving the clinical site to allow management of any potential adverse events.

#### Follow-up contact

The follow-up assessment will be performed by a phone call to the subjects between 5 and 7 days after discharge. The PI will obtain information on any new AEs and new/changes to concomitant medication since discharge and complete the corresponding pages of the eCRF. Provided there are no AEs which require further attention, the subject’s participation in the study will be complete. A visit will be scheduled if deemed necessary by the PI or their suitably qualified designee.

Subjects who, after randomisation, discontinue the trial prematurely should be encouraged to participate in this follow-up procedure. If their withdrawal is due to safety evaluations, then the subjects will be followed until these return to baseline levels or until the PI has determined that these events are no longer clinically significant.

Subjects who develop an AE at any time during the study will be followed until any required evaluations have returned to baseline or until the PI has determined that these events are no longer clinically significant. Reported AEs will be followed until resolution whenever possible.

#### Study termination

If, in the opinion of the PI, the clinical observations in the study suggest that it may be unwise to continue, the PI may terminate part of or the entire study after consultation with the Sponsor. In addition, the Sponsor may terminate part of or the entire study for safety or administrative reasons. A written statement fully documenting the reasons for study termination will be provided to the IRB.

### Assessments

#### Demographic data

Socio-demographic data (sex, age) will be recorded at screening.

#### Smoking history and willingness to quit smoking

Subjects will be asked about their smoking history at screening. This will include questions to evaluate whether the subject has smoked for at least the last 3 consecutive years, to determine the number of cigarettes smoked by the subject per day on average, to check that the subject has smoked their chosen brand of conventional cigarette consistently for at least 6 months, and to determine if the subject regularly uses any nicotine or tobacco product other than commercially manufactured filter cigarettes. In addition, the subject will be asked if he/she is planning to quit smoking within the next 12 months. A colour photocopy of the subjects preferred cigarette pack, including the surface showing tar/nicotine content, will be taken.

#### Medical history, concomitant diseases, and concomitant medication

Relevant medical history, as determined by the PI or their suitably qualified designee, will be documented at screening and admission. Medical history will include any clinically significant neurological, gastrointestinal, renal, hepatic, cardiovascular, psychiatric, respiratory, metabolic, endocrine, haematological or other major disorders. Medical history is defined as any condition that started and ended prior to screening. A concomitant disease is any disease that started prior to, and which was still ongoing, at screening.

Prior medications taken within 28 days prior to screening and any concomitant medication will be documented at screening and admission. Any medication which was started prior to screening and is still being taken by the subject, or any medication started after screening, will be considered to be a concomitant medication. This includes both prescription and OTC products.

Details of prior and concomitant medications to be recorded include the drug name (generic and trade name if applicable), route of administration, total dose/unit, indication, start date and stop date.

### Vital signs

Supine blood pressure, supine pulse rate, respiratory rate and axillary body temperature will be measured at screening, admission, and discharge. Vital signs will also be performed at other times if judged to be clinically appropriate.

Blood pressure and pulse rate will be measured using automated monitors. Subjects will be supine for at least 5 min before blood pressure and pulse rate measurements. Respiratory rate will be measured as per the standard institutional practice. Axillary body temperature will be measured singly using a digital thermometer.

### Physical examination

A full physical examination will be performed at screening, and an abbreviated physical examination will be performed at admission and discharge.

### Height, body weight and body mass index

Height in metres (to the nearest centimetre) will be measured at screening, and weight in kilograms (to the nearest 0.1 kg in light indoor clothing and without shoes) will be measured at screening, admission, and discharge.

The body mass index (BMI) at screening will be calculated using the following formula:

$$ \mathrm{BMI}=\frac{\mathrm{body}\  \mathrm{weight}\ \left(\mathrm{kg}\right)}{{\left[\mathrm{height}\ \left(\mathrm{m}\right)\right]}^2} $$

### Electrocardiography

A single 12-lead resting ECG with a 10-s rhythm strip will be recorded after the subject has been supine for at least 5 min at screening and discharge (or early termination). The 12-lead ECG will be repeated once if either of the following criteria apply:

- The QT interval corrected for heart rate (QTc) is >500 ms.
- The QTc change from the baseline (pre-product use) is >60 ms.

If repeated, the repeat values will be used for data analysis. Additional 12-lead ECGs will be performed at other times if judged to be clinically appropriate or if the ongoing review of the data suggests a more detailed assessment of ECGs is required. A physician will perform a clinical assessment of each 12-lead ECG. Clinical site reference ranges will be applied to all ECG parameters determined throughout the study. The ECG machine will compute the PR and QT intervals, QTc, QRS duration and heart rate. The QT interval will be corrected for heart rate using Bazett’s formula (QTcB).

#### Clinical laboratory tests

Blood and urine samples will be collected for clinical laboratory evaluations at screening, admission, and discharge (see Table 6 for details). Additional clinical laboratory evaluations will be performed at other times if judged to be clinically appropriate or if the ongoing review of the data suggests a more detailed assessment of clinical laboratory safety evaluations is required. The PI will perform a clinical assessment of all clinical laboratory data.

**Table 6:** Clinical laboratory tests

Serum biochemistry	Haematology
Aspartate aminotransferase (AST)	Haemoglobin
Alanine aminotransferase (ALT)	Haematocrit (packed cell volume [PCV])
Alkaline phosphatase	Total and differential leukocyte count
Gamma-glutamyl transferase (GGT)	Red blood cell (RBC) count
Sodium	Platelet count
Potassium	Mean cell volume (MCV)
Chloride	Mean cell haemoglobin (MCH)
Calcium	MCH concentration (MCHC)
Inorganic phosphate	Urinalysis:
Glucose	Microscopic examination
Urea	pH
Uric acid	Specific gravity
Total bilirubin	Protein
Direct bilirubin	Glucose
Creatinine	Ketones
Total protein	Bilirubin
Albumin	Blood
Total cholesterol	Nitrite
Triglycerides	Urobilinogen
Creatinine phosphokinase (CPK)	Leukocytes
Blood urea nitrogen (BUN)	Urine pregnancy test^d^
Serology^a^	Urine drug screen^e^
Hepatitis B surface antigen (HBsAg)	Additional Tests
Hepatitis C antibody	Alcohol breath test
Human immunodeficiency virus (HIV)^c^ Syphilis	
Hormone Panel^b^	
Follicle-stimulating hormone (FSH)	
Human chorionic gonadotropin (hCG; serum pregnancy test)	

^a^Screening only

^b^In all females at screening only

^c^HIV1/2 and p24 antigen

^d^In all females. A positive urine pregnancy test will be confirmed with a serum pregnancy test

^e^Urine drugs of abuse screen will be conducted for amphetamines, barbiturates, benzodiazepines, cocaine, ecstasy, methamphetamine, morphine, methadone, tricyclic antidepressants, and tetrahydrocannabinol

### Urine drugs of abuse screen and alcohol breath test

Subjects will be asked to provide urine samples for a drugs of abuse screen at screening and admission (Day −1). Urine samples will be screened for the presence of the following drugs of abuse: amphetamines, barbiturates, benzodiazepines, cocaine, ecstasy, methamphetamine, morphine, methadone, tricyclic antidepressants, and tetrahydrocannabinol. Subjects will be screened for the presence of breath alcohol.

#### Urinary biomarkers of exposure and of biological effect

Urine samples will be collected over consecutive 24-h time periods, beginning on the evening (between approx. 19:00 and 20:00) of admission (Day −1), with the last time period finishing on the evening (between approx. 19:00 and 20:00) on Day 7. All urine voided will be collected. The samples will be refrigerated at between 2 °C to 8 °C.

The urine samples collected during a single 24-h interval will be pooled together at the end of the 24-h period and weighed. The samples will be related to the day on which the 24-h collection period ended. The pooled samples will be thoroughly mixed before providing aliquots for analyses. The total volume of all urine collected over each 24-h period will be calculated from weight and specific weight. Urine aliquots will be stored in suitably labelled tubes at −18 °C to −25 °C pending assay.

The urinary BoE and BoBE that will be analysed are presented in Table 1. Creatinine in urine will also be calculated and used to report BoBE as amount per unit of creatinine. The independent laboratories performing the analysis of BoE and BoBE will be blind to each subject’s test product allocation.

#### Blood sampling for white blood cell count

Blood samples for white blood cell count will be collected in 2 appropriately sized EDTA tubes at approximately 16:00 on Day 2, Day 5 and Day 7 using direct venepuncture.

#### Nicotine pharmacokinetic assessments

Subjects in Groups A to E will be required to abstain from using any nicotine products from the evening of Day 7 (after the last 24 h urine sample), until the morning of Day 8 (at least 12 h of smoking cessation). Subjects will then be required to smoke either 1 conventional cigarette or use THP with 1 tobacco stick for up to 5 min, dependent on their study group. Blood samples (13 × 4 mL) will be taken by direct venepuncture or from a cannula placed in a forearm vein, at 5 (±2) minutes before smoking, and at 1 (±0.5), 3 (±0.5), 4 (±0.5), 5 (±0.5), 6 (±0.5), 7 (±0.5), 10 (±1), 15 (±1), 30 (±5), 60 (±5), 120 (±10), and 240 (±10) minutes relative to first puff. The concentration of nicotine will be measured in all plasma samples.

During the single product use in the pharmacokinetic assessment period (Day 8), the number of individual puffs taken by the subject on either a conventional cigarette or on the assigned THP product will be recorded by the clinical site staff in the eCRF.

#### Exhaled carbon monoxide

Exhaled carbon monoxide (eCO) levels will be measured at Screening, Admission and at the same time each evening (between approx. 16:30 and 17:30) from Day 1 to Day 7. The date and time of the CO measurement along with the eCO in parts per million will be recorded on the source documents and entered on the eCRF. Each eCO measurement will take place a minimum of 30 min after the subject’s previous use of any tobacco product.

#### Pulmonary function

Spirometry with and without a short-acting bronchodilator (sultanol) will be conducted at screening, admission and discharge/early termination to evaluate inclusion/exclusion criteria (the post-bronchodilator results). At screening, spirometry without bronchodilator will be conducted first, followed by spirometry with bronchodilator. Also at screening, spirometry will be conducted at least 1 h after smoking. Spirometry will be used to measure forced vital capacity (FVC), forced expiratory flow 25–75% (FEF_25–75%_), peak flow, and forced expiratory volume in 1 s (FEV_1_).

Spirometry will be conducted using the guidelines specified by the Third National Health and Nutrition Examination Survey (NHANES III; [10]). Spirometry testing will be performed in accordance with procedures of the American Thoracic Society/European Respiratory Society [21]. Predicted FEV_1_ and FVC will be calculated according to the formula recommended by the Japanese Respiratory Society.

Measurements will be repeated if there are technical issues during testing or if the subject shows signs of fatigue. Measurements will not be repeated if the results fall under the levels required for enrolment in the study. If a subject shows signs of fatigue during repeated testing, testing will be halted to ensure that the subject does not become exhausted and will only be recommenced at the physician’s discretion. No more than 8 manoeuvers will be performed. The spirometry traces will be copied and inserted into the source documents. The spirometer will be kept calibrated as recommended by the manufacturer.

### Blood samples

Blood samples will be collected by qualified and trained site personnel. The maximum total volume of blood drawn for each subject will be 150 mL. The blood volumes shown in Table 7 will be withdrawn for each subject. Blood samples will be destroyed as per the laboratory’s standard procedures.

**Table 7:** Blood samples

	Volume per blood sample (mL)	Maximum number of samples	Total amount of blood (mL)
Safety lab tests	7.5	3	22.5
Serology	3.5	1	3.5
WBC count	5	3	15
Pharmacokinetics (Groups A-E only)	4	13	52
		Total (Groups A-E):	93
		Total (Group F):	41

#### Product consumption

The number of conventional cigarettes smoked or THP sticks used will be recorded over continuous 24-h periods (concurrent with urine collection periods) from the evening of Day −1 until the beginning of the exposure period (Group F) or the evening of Day 7 (Groups A to E).

### Questionnaires

A paper copy of the subject questionnaires used in this study will be completed by each subject, and the PI/site staff will review each questionnaire for completeness. Subjects will be required to answer all questions on each questionnaire. Questionnaires will be provided in Japanese.

#### Fagerström test for cigarette dependence (FTCD)

Potential nicotine dependence will be assessed via a questionnaire at Screening using a FTCD questionnaire in its revised version ([6]; Table 8). The questionnaire consists of 6 questions which will be answered by the subject himself/herself. The scores obtained on the test permit the classification of nicotine dependence into 3 levels: Mild (0–3 points), moderate (4–6 points), and severe (7–10 points).

**Table 8:** Fagerstrom Test for Cigarette Dependence

**1.** How soon after you wake up do you smoke your first cigarette?
□ Within 5 min
□ 6–30 min
□ 31–60 min
□ After 60 min
2.Do you find it difficult to refrain from smoking in places where it is forbidden?
□ Yes
□ No
3.Which cigarette would you hate most to give up?
□ The first one in the morning
□ All others
4.How many cigarettes/day do you smoke?
□ 10 or less
□ 11–20
□ 21–30
□ 31 or more
5.Do you smoke more frequently during the first hours after waking than during the rest of the day?
□ Yes
□ No
6.Do you smoke if you are so ill that you are in bed most of the day?
□ Yes
□ No

#### Product satisfaction

A product satisfaction question will be administered to subjects during the pharmacokinetic assessment period (Day 8) between the 10-min and 15-min blood draws. The question, “Can you tell me how much do you like this tobacco product?”, will be answered by the subject himself/herself. The subject will provide responses on a 7-point Likert scale ranging from “1 – I dislike it a lot” to “7 – I like it a lot” (scores of 2–6 will not have a descriptor).

### Determination of sample size

A sample size of 30 subjects per group has been set for this study. This is based on powering the primary objective of within-group comparison of biomarker levels at baseline and end-of-study. The calculation was based on the number of pairs required to perform a paired t-test with 80% power for a decrease in biomarker levels of 40% or more compared to historical biomarker data available for a 7 mg ISO tar conventional cigarette ([4], [31]). A sample size of 30 was determined to be adequate based on the biomarker requiring the most pairs to power (eCO) and allowing for attrition. A sample size of 30 should also provide sufficient power for the secondary objective of between-group comparisons, based on a minimum of 40% reduction in BoE.

A number of subjects greater than those required to complete the study will be screened. Furthermore, at the start of each clinic visit, some subjects will act as ‘reserves’ and will enter the study should the required number of subjects not attend the clinic. These measures will ensure that the required number of subjects who meet all inclusion/exclusion criteria are both recruited onto and enter the clinical phase of the study such that the target sample size is met.

### Statistical analysis

Statistical analyses will be performed by Covance. A detailed statistical analysis plan will be prepared before database closure and agreed by the sponsor and Covance (Additional file 2: Appendix 2). Any changes in the planned statistical methods will be documented in the clinical study report.

The primary objective will be examined by computing levels of biomarkers at baseline, and at the end-of-study. These data will be compared within respective groups using appropriate statistical test methods. Data will be examined and may be transformed to ensure that any assumptions associated with particular statistical tests or models are obeyed.

Similarly, biomarker measures in the secondary objectives will be examined by computing levels of biomarkers at baseline and at the end-of-study. Appropriate statistical tests and comparisons will be made between groups. Within-group comparisons will be conducted for all groups. For between- group comparisons, the following groups will be compared:

- Group A to Group B
- Group A to Group E
- Group B to Group F
- Group C to Group D
- Group D to Group F
- Group A to Group F
- Group C to Group F

Continuous variables will be presented by means of descriptive statistics (N, mean, standard deviation, median, minimum and maximum) and will be calculated for the BoE, BoBE and nicotine pharmacokinetic parameters. Categorical variables will be displayed by means of frequency tables and, where appropriate, shift tables. All summaries will be presented by study arm, sex, age, and timepoint.

The statistical analysis will be based on separate, hierarchically-organised, analysis populations defined as the following:

- **Safety population** - All subjects who smoked at least one cigarette or had at least one safety assessment after enrolment on Day −1.
- **Intent-to-treat population (ITT)** - All subjects who were assigned and had at least one valid assessment of a biomarker variable.
- **Per-protocol population (PP)** - All subjects who had valid assessment of a biomarker variable and completed study according to the protocol (no major protocol deviations).
- **Pharmacokinetic population** - All subjects who had had sufficient data to calculate at least 1 pharmacokinetic parameter and completed study according to the protocol (no major protocol deviations).

All safety data will be summarised for all safety parameters based on the safety population,

stratified by study arm.

### Data management

Full data management procedures will be contained in a Data Management Plan provided by Covance. This will be provided before study commencement.

### Adverse events management

The condition of each subject will be monitored from the time of signing the ICF, to final Discharge from the study; including the Follow-up period. In addition, any signs or symptoms will be observed and elicited at least once a day by open questioning, such as “How have you been feeling since you were last asked?” Subjects will also be encouraged to spontaneously report AEs occurring at any other time during the study.

Any AEs and remedial action required will be recorded in the subject’s source data. The nature, time of onset, duration and severity will be documented, together with the PI’s opinion of the relationship to product use. Any clinically significant abnormalities identified during the course of the study will be followed-up until they return to normal or can be clinically explained.

Any AE assessed as related to the investigational products (IP) will be assessed for its expectedness. An AE will be regarded as ‘unexpected’ if its nature or severity is not consistent with information already known about the IP, and is not listed in the current Investigator’s Brochure (IB). The IB provides further detail on signs or symptoms that might be expected with the use of the IP, including information relating to malfunction or misuse.

The PI or their appropriately qualified designee will review each event, grade its severity and assess its relationship to the product consumption and/or the study procedures. The date of onset, time of onset, and outcome of each event will be noted. If any of the above AEs are serious, special procedures will be followed. The Investigator will report all serious adverse events to the Covance Pharmacovigilance and Drug Safety Services Department within 24 h. Written reports will then be submitted within 48 h, whether or not the SAEs are deemed related to study procedures or products. The IRB will be notified by these reports. If an SAE occurs during this study, the study Medical Monitor will also be contacted.

### Assessing study conduct

Quality control and quality assurance will be performed according to Covance standard operating procedures (SOPs) or per Sponsor request and as applicable according to the contract between Covance and the Sponsor.

The Sponsor will designate an independent CRA to perform the duties of a Study Monitor and who will be responsible for monitoring this clinical study. The Study Monitor will monitor the study conduct, eCRF and source documentation completion and retention, and accurate study IP accountability. To this end, the Study Monitor will visit the study site at suitable intervals and be in frequent contact through verbal and written communication. It is essential that the Study Monitor has access to all documents, related to the study and the individual subjects, at any time these are requested. In turn, the Study Monitor will adhere to all requirements for subject confidentiality as outlined in the ICF.

### Bioanalytical methods

The BoE selected as endpoints in this study are based around the initial list of priority toxicants proposed by the WHO Study Group on Tobacco Product Regulation (TobReg), and also selected based on the availability of robust analytical methods for their measurement and their indication of exposure to a range of both gas phase and particulate cigarette smoke toxicants.

The two BoBE selected for evaluation are markers for oxidative stress and inflammation, which may be reduced acutely in smokers who either switch products or quit smoking. In all bioanalysis, the analytical laboratories will be blinded to the subjects’ test products by the use of a sample labelling system which identifies samples to the laboratories by unique identifier numbers only. Exhaled CO levels will be measured using the EC50 Micro III Smokerlyzer (Bedfont) CO meter, or similar device (Bedfont Scientific Ltd., Maidstone, UK). White Blood Cell Count will be analysed using methods adapted from Haswell et al. [11]. Analysis will be carried out at LSI Medience Corporation, 36–1 Shimizu-cho, Itabashi-ku, Tokyo.

The following analyses will be carried out at Analytisch-biologisches Forschungslabor (ABF) Goethestraße 20, 80,336 München, Germany:

- Total NNAL and total NNN methods will be adapted from [15, 16]
- All mercapturic acids (acrolein, crotonaldehyde, benzene, 1,3-butadiene, acrylonitrile, ethylene oxide, 2-MHBMA and acrylamide) will be analysed using methods adapted from Pluym et al. [25].
- Aromatic amines (4-aminobiphenyl, 2-aminonaphthalene and o-toluidine) will be analysed using methods adapted from Feng et al. [8] and Riedel et al. [26].
- Creatinine will be analysed as described in Blaszkewicz et al. [2].

The following analyses will be carried out at Celerion Inc., 621 Rose Street, Lincoln, Nebraska 68,502, USA:

- Total nicotine equivalents (T_neq_; nicotine, cotinine, 3-hydroxycotinine and their glucuronide conjugates) will be analysed using methods adapted from Piller, M., et al. [24].
- 1-hydroxypyrene will be analysed using methods adapted from Shepperd et al. [29].
- 8-epi-prostaglandin F2α Type III will be analysed using methods described in Haswell et al. [11].
- Plasma nicotine will be analysed using methods described in Fearon et al. [7].

## Discussion

The development and marketing of novel tobacco products with, relative to conventional cigarettes, reduced levels of toxicants in their emissions is a potential way to reduce the harms associated with cigarette smoking [32]. We have previously demonstrated exposure reductions in smokers who switch to smoking cigarettes with modified tobacco blend and physical characteristics [4, 30, 31]. The current study primarily aims to objectively quantify any changes in exposure when smokers switch to using a THP compared to those who remain smoking conventional cigarettes. Data from this study are expected to increase our understanding of toxicant exposure in users of novel tobacco products and the results will be published in peer-reviewed scientific journals.

Should any such reduction in exposure be observed, this cannot be extrapolated to a reduction in harm or risk and our previous work has shown that significant reductions in exposure had little or no effect on BoBE [31]. However, such an exposure change may be used to add support to a weight of evidence approach to providing a more global assessment of the relative individual risks of using a THP compared to smoking a cigarette, and this would support a follow-up study utilising a broader suite of BoBE to gain an understanding of any risk reduction as a consequence of the change in exposure.

This study will also allow us to determine nicotine pharmacokinetic parameters for the THPs. While such data are readily available for cigarettes and electronic cigarettes few studies have been reported describing nicotine delivery from THPs. Such data are important since effective nicotine delivery is considered to be a potential determinant of both the uptake and continued use of alternatives to cigarette smoking by current smokers [7, 27].

### Limitations

The data obtained in this study may be limited by various factors. Firstly, any observed changes in BoE and BoBE could be limited to the types of subject recruited into this study. Daya may not easily be extrapolated into other populations (eg. Westerners) or cohorts (eg. those with a different smoking history or who exhibit different smoking behaviours than those who are recruited into this study). Secondly, although this study will examine a comprehensive suite of BoE the data obtained may not be representative of all smoke chemicals to which smokers may be exposed. Thirdly, due to nature of this short-term clinical confinement study, data obtained may not be presentative of those seen in a real-world (ambulatory) setting over a prolonged period. Finally, although this study will examine a single BoBE, this study will not be able to discern whether switching to any of the study products will have any beneficial health effects or to determine whether any of the test products used will present a lesser risk of smoking-related disease than continued smoking.

## Additional files

Additional file 1: Appendix 1: Informed Consent Form. (DOCX 78 kb)

Additional file 2: Appendix 2: Statistical Analysis Plan. (DOCX 96 kb)

## Abbreviations

1-OHP: 1-hydroxypyrene
3-HPMA: 3-hydroxypropylmercapturic acid
4-ABP: 4-aminobiphenyl
8-Epi-PGF2α Type: III 8-epi-prostoglandin F2α type iii
AAMA: N- Acetyl-S-(2-carbamoylethyl)cysteine
AE: Adverse event
BMI: Body mass index
BoBE: Biomarker of biological effect
BoE: Biomarker of exposure
CEMA: 2-cyanoethylmercapturic acid
CO: Carbon monoxide
CRA: Clinical Research Associate
CRO: Contract research organisation
CYP: Cytochrome P450
ECG: Electrocardiogram
eCO: Exhaled carbon monoxide
eCRF: Electronic case report form
EDTA: Ethylenediaminetetraacetic acid
FEV_1_: Forced expiratory volume after 1 s
FTCD: Fagerström test for cigarette dependence
FVC: Forced vital capacity
GAMA: N-acetyl-S-(2-hydroxy-2-carbamoylethyl)cysteine
HEMA: 2-hydroxyethylmercapturic acid
HMPMA: 3-hydroxy-methylpropylmercapturic acid
IB: Investigator’s Brochure
ICF: Informed consent form
ICH: International Conference on Harmonisation
IRB: Institutional review board
ISO: International Organisation for Standardisation
MHBMA: Monohydroxybutenyl-mercapturic acid
MRTP: Modified risk tobacco product
NNAL: 4-(methylnitrosamino)-1-(3-pyridyl)-1-butanol
NNN: N-nitrosonornicotine
OTC: Over-the-counter
PI: Principal Investigator
QTc: QT interval corrected for heart rate
S-PMA: S-phenylmercapturic acid
THP: Tobacco heating product
TNeq: Total nicotine equivalents
WBC: White blood cell
WHO: World Health Organisation
