# Supplementary material for: A randomised, controlled, two-Centre open-label study in healthy Japanese subjects to evaluate the effect on biomarkers of exposure of switching from a conventional cigarette to a tobacco heating product
Source: BMC Public Health. 2017 Aug 22;17:673. doi: 10.1186/s12889-017-4678-9 (PMC5567437; doi:10.1186/s12889-017-4678-9)
Supplement: Supplementary file 1 — Appendix 1: Informed Consent Form. (DOCX 78 kb) [file 12889_2017_4678_MOESM1_ESM.docx]

**Appendix 1.**

**Informed Consent Form.**

INFORMED CONSENT FORM

| Study Title: | A Randomized, Controlled, Multi-centre Open-label Study in Healthy Japanese Subjects to Evaluate the Effect on Biomarkers of Exposure of Switching from a Conventional Combustible Cigarette to the Glo Tobacco Heating Product. |
| --- | --- |
| Protocol No.: | BAT3416008 |
| Name and Address of Sponsor: | British American Tobacco (Investments) Ltd.  R&D Centre Regents Park Rd.  Southampton  Hampshire SO15 8TL, UK |
| Principal Investigator: | *<Insert PI Name and title) (use Medical Doctor for title)* |
| Telephone: | *<Insert numbers including 24h contact number>* |
| Research site Name/Address: | *<Insert research site name and address>* |

**It is important that you give a true and complete medical history. You must be honest about your past and present usage of medications. Giving information that is not true could be very harmful to your health. If you give false information, you may be dismissed from the study.**

You are being asked to take part in a research study sponsored by British American Tobacco (Investments) Limited (Sponsor). The clinical research site and third party vendors, Covance and CMIC, are being paid by British American Tobacco (Investments) Limited to conduct this study. You should read this form before you decide if you want to take part in the study. This form will tell you about the study.

The study Doctor or study staff can explain words or information that you do not understand. Ask the study staff as many questions as needed for you to decide if you want to take part in the study.

Research studies are voluntary and include only those who wish to take part. If you decide to take part in this study, you must sign your name at the end of the form and date it. You cannot take part in this study until you sign and date this form.

Once you have signed and dated this Informed Consent Form, in the presence of the Investigator, you will receive one original which you will take home. When making the decision to participate in the study, it is important that you accept its course, its purpose, the planned procedures, and that you are ready to participate until the end of the study.

Prior to the start of this study, the institutional review board has reviewed the scientific and ethical aspects of the study and given their approval.

Institutional Review Board:

Prior to the start of this study, the institutional review board established in the hospital has reviewed the ethical and scientific validity of the study and the appropriateness of the conduct of the study in the hospital, and given their approval. The institutional review board will also review the appropriateness of continuing the study when necessary. For more information about written procedures and membership lists of the committee, summaries of minutes of its meetings, etc., please contact the research site study office. You can also find such information on the web of the committee.

- Name: Hakata Clinic Institutional Review Board
- Person responsible for establishment: Masanari Shiramoto
- Address: Random Square, 5-7 FL 6-18 Tenyamachi, Hakata-Ku, Fukuoka, Japan, 812-0025
- Website URL: <http://www.lta-med.com/SouseikaiGlobal/irb.php>

1. **INTRODUCTION**

Smoking is a leading cause of numerous illnesses including lung cancer, chronic obstructive lung disease and heart disease. There are no safe tobaccos, and the best way for smokers to reduce health damage is to stop smoking. The health risks associated with cigarette smoking are known to be due to chemicals in cigarette smoke which are toxic and can lead to changes in the body, causing disease. Nicotine is primarily responsible for the addictive properties of cigarette smoking.

Tobacco Heating Products (THP) consist of a heating device which heats rather than burns a stick of tobacco. This stick of tobacco is similar in appearance to a cigarette. The Sponsor’s own assessments of the chemicals found in the vapour from a THP when puffed on a puffing machine show significant reductions in the levels of many chemical toxicants when compared to those found in cigarette smoke. It has been seen that this vapour contains significant levels of nicotine.

Overall, the aim of this study is to help the Sponsor to understand whether a smoker who switches to using a THP would see reductions in their exposure to toxic chemicals associated with cigarette smoking.

Participants in this study are required to understand that there are no “safe” tobaccos. The only known way to reduce the risks of smoking-related diseases (e.g. cancers, lung, blood or heart diseases) is to stop smoking.

1. **PURPOSE OF THIS STUDY**

This study was designed for research purposes to collect the data on the Sponsor’s newly developed Glo THP in adult smokers. The main purposes of this study are:

- To determine whether the body is exposed to a lower amount of certain toxic chemicals in vapour from THPs as compared to the smoke from regular cigarettes
- To compare how nicotine is absorbed in the body from smoking different THPs with regular cigarettes

Reductions in the level of exposure to toxic chemicals are measured in your biomarkers. A biomarker of exposure is a measure of a substance generated in your body when you absorb certain materials, for example, cigarette smoke. For example, nicotine is broken down in the body into several chemicals including cotinine and 3-hydroxycotinine. By measuring the amount of these chemicals in urine, the total exposure to nicotine can be calculated. We will also measure other biomarkers in a similar way.

1. **TEST METHODS**

Tobacco Heating Products (THPs) have been recently developed as an alternative to conventional cigarettes. A THP is a tobacco product that heats tobacco (typically to temperatures around 240 to 350ºC) rather than combusting (burning) it. THPs consist of a heating device which heats a stick of tobacco that is similar in appearance to a cigarette.

3.1 Test Products

As part of the study, you will be asked to use some of the following products, depending on which study group you are assigned to:

- Product A – Regular combustible cigarette
- Product B – Glo with tobacco ‘Neostik’ consumable
- Product C - Menthol combustible cigarette
- Product D– Glo with menthol tobacco ’Neostik’ consumable
- Product E – Commercially-available THP with tobacco consumable

Product A and C – Regular and Menthol combustible and commercially available cigarettes (7 mg/cig ISO tar) will be provided by the Sponsor.

Product B and D - The Glo device has been designed and manufactured for the Sponsor. The Glo tobacco heating product consists of a tobacco ‘Neostik’ that resembles a regular tobacco cigarette and an electronic heating device. The heating device is comprised of a rechargeable battery, an electrical element which heats the Neostik and electronic circuitry which controls the warming up, heating temperature and heating period of the device.

Product E – Commercially-available THP with tobacco consumable is a tobacco heating product not designed or manufactured for the Sponsor but already commercially available on the Japanese market.

Because this is a research study, the study products will be given to you only during this study and not after the study is over.

3.2 Test Methods

This study is a randomized, open label study comparing effects of switching from smoking conventional cigarettes to use of the Glo THP, with continued smoking of conventional cigarettes or smoking cessation. In total this study will enroll approximately 180 subjects.

You will be randomly assigned to one of 6 study groups (to be labelled A, B, C, D, E and F). If you are assigned to the conventional cigarette group, Glo group or an alternative THP group (study groups A to E), you can smoke the product assigned to you up to a maximum limit based on your usual daily consumption before you joined the study. You will be informed of your maximum limit by the study coordinator. As a rule you will be asked not to smoke between the hours of 00:00 and 06:00 during your stay at the research site. However, if you are assigned to the smoking cessation group (study group F), you will need to refrain from use of all nicotine / tobacco containing products for 5 days.

You will participate in this study for up to 50 days. This period includes a screening period (of up to 34 days before admission), 8 or 9 days as an inpatient staying at the clinic (from check in on Day -1 to the day of discharge), and a follow-up period of between 5 to 7 days from the day of discharge.

Screening Period (up to 34 days before admission)

The Screening period is within 34 days before admission for the inpatient visit and consists of a telephone call and visit to the research site. Following the telephone screening you will be asked to visit the research site for additional Screening tests. Prior to the Screening visit you will need to refrain from strenuous physical activity (beyond your normal activity levels) for 7 days, abstain from alcohol for 72 hours and avoid eating poppy seeds for 3 days.

Inpatient Period (from admission to discharge)

If you are assigned to either Group A, B, C, D or E then you will stay in the research site for 9 consecutive days. If you are assigned to Group F then you will stay in the research site for 8 consecutive days.

- Baseline Period (Day -1 to evening of Day 2)

Following completion of all other admission tests, including confirmation of negative urine pregnancy test, you will be offered a THP product test of up to 3 sticks (Glo THP and commercially-available THP non-menthol, or Glo THP menthol, based on whether your usual cigarette is a regular or menthol type). Also based on whether your usual cigarette is a menthol or regular type, you will then be randomly assigned to a study group by the research site staff. From the time of check in to the research site on Day -1 to the evening of Day 2 you will be asked to smoke a menthol or non-menthol version of the supplied conventional cigarette (to match your usual type). Throughout the study you must return all used and unused cigarettes, filter tips, THP devices and THP consumables to the research site staff following use. You must not take any products home with you at the end of the study.

- Exposure Period (evening of Day 2 to evening of Day 7)

On the evening of Day 2 you will stop smoking the supplied cigarettes and move to your assigned study group. From then on you will use the product assigned to your group (or stop smoking if you are assigned to the cessation group):

- - Group A: you will continue to smoke non-menthol conventional cigarette
  - Group B: you will use Glo THP with Neostik
  - Group C: you will continue to smoke menthol conventional cigarette
  - Group D: you will use Glo THP with menthol Neostik
  - Group E: you will use the commercially-available THP with tobacco consumable
  - Group F: you will stop smoking

If you are a menthol smoker, you will only be assigned to use a menthol product (Groups C or D). If you are a non-menthol smoker, you will only be assigned to use a non-menthol product or to stop smoking (Groups A, B, E or F). At the end of the Exposure period, Group F will be discharged from the study and will enter the Safety follow-up period. Groups A, B, C, D and E will move to the Pharmacokinetic Assessment Period.

- Pharmacokinetic Assessment Period (evening of Day 7 to discharge on Day 8)

On the evening of Day 7 you will be asked to refrain from smoking for 12 hours until the morning of Day 8. On the morning of Day 8 you will be asked to smoke either a single conventional cigarette (Groups A and C) or use your assigned THP (Groups B, D and E) for a maximum of 5 minutes. Blood samples will be collected before, during and after this smoking period. You will also be asked a question about your satisfaction with the product you are assigned to use in this session.

Following this Pharmacokinetic Assessment period you will be discharged from the study and will enter the Safety follow-up period.

- Discharge from study

Group A, B, C, D and E will discharge from the study on Day 8 following completion of the pharmacokinetic Assessment Period. Group F will discharge from the study on Day 7 following completion of the Exposure period. If you wish to return to smoking your usual cigarette, you may smoke your first cigarette before leaving the research site.

- Safety follow-up period (up to 7 days after discharge)

You will enter the Safety follow-up period for up to 7 days after discharge. During this time, if you feel unwell or feel any abnormalities in your body, please contact the research site on the 24 hour telephone number that you have been provided with.

The design of this study is shown as follows:

Smoking history and study requirements

Only smokers who smoke commercial menthol or non-menthol type cigarettes containing between 6mg and 8mg of ISO tar can participate in this study. You will have smoked regularly for at least 3 years prior to your screening visit and between 10 and 30 cigarettes per day. Whilst on the study, during the baseline and exposure periods, you will be allowed to smoke no more than 120% of your usual number of cigarettes per day. The study coordinator will maintain a record of the number of cigarettes that you smoke and, if you reach your limit, no further cigarettes will be allocated to you. Smoking is permitted at the designated times (6:00 to 00:00) during your stay in the research site. In addition you cannot smoke during other test procedures. During the designated smoking time it is your choice when you smoke but you cannot smoke without asking the research site staff. You may only smoke in a designated smoking room. When you want to smoke, please let the person in charge know every time. The person in charge will hand your assigned smoking product to you directly, every time.

During the Baseline period, you will be given a conventional cigarette to smoke. During the Exposure period, you will change to your assigned study group; conventional menthol or non-menthol cigarettes, Glo with Neostik, commercially-available THP with tobacco consumable, or the smoking cessation group.

If you are assigned to the smoking cessation group you will be instructed to refrain from using any nicotine/tobacco containing products from approximately 19:00 on Day 2 until your release from the research site on the evening of Day 7, and you will not be allowed to enter the smoking room. The study doctor will be available to support you if you are experiencing symptoms of nicotine withdrawal, however no smoking cessation aids will be provided. If you decide to smoke conventional cigarettes during this time, you must discontinue participation in this study following consultation with the study doctor.

If you are assigned to the Glo or commercially-available THP group, you cannot smoke a conventional cigarette from approximately 19:00 on Day 2 until approximately 13:00 on Day 8. If you decide to smoke conventional cigarettes during this time, you must discontinue participation in this study following consultation with the study doctor. You will also be instructed to refrain from using any nicotine or tobacco products for a minimum of 12 hours from approximately 19:00 on Day 7 until the morning of Day 8 when you will participate in the pharmacokinetic assessment period.

You may decide to quit smoking, or to withdraw from the study, at any time. If you do wish to stop smoking, you can be appropriately supported by the study doctor and research site team. If you are assigned to the conventional cigarette group, Glo or commercially-available THP and you decide to quit smoking / using the THP during the baseline period or the exposure period then your study participation will be discontinued.

Dietary restriction

During your stay at the research site all your meals will be provided and the consumption of water is permitted.

To avoid effects on the measurement of your biomarkers of exposure, you are required to avoid certain foods for 48 hours prior to your admission. You must avoid eating or being in the presence of the cooking of cruciferous vegetables, grilled, smoked, fried or barbecued food. You must avoid eating poppy seeds for 3 days prior to your screening and admission visit. You must refrain from using alcohol for 3 days prior to admission.

You cannot bring your own food or drink of any description to the research site during the study period.

Medication

If you require medication during the study (from the screening period to completion of the study), please consult the study doctor at the Screening session and prior to use. Your study doctor will be responsible for your care whilst you are participating in the study.

3.3 Scheduled duration of your participation in the study.

You will participate in this study for up to 50 days.

- Screening period: up to 34 days
- Study period in research site: 8 days (smoking abstinence group) or 9 days (cigarettes, Glo or commercially-available THP)
- Safety follow-up period: 5 to 7 days

3.4 Scheduled participation in the study.

180 persons are scheduled to participate in the study. The rate of inclusion to each study group is as follows:

- Group A – Conventional Cigarette – non-menthol – 30 subjects
- Group B – Glo THP with Neostik – non-menthol - 30 subjects
- Group C – Conventional Cigarette – menthol – 30 subjects
- Group D – Glo THP with Neostik – menthol – 30 subjects
- Group E – Commercially-available THP with tobacco consumable – non-menthol – 30 subjects
- Group F – Smoking Abstinence – 30 subjects.

3.5 Discontinuation of participation in the study

You can leave the study at any time. If you want to end your participation in the study, you will be able to do so unconditionally. Please inform the study doctor or the person in charge. If you discontinue the study you will be asked for a reason but you don’t have to answer if you don’t want to.

In addition, we will discontinue your participation in the study in the following cases. In these cases we may discontinue your participation in the study even though you may want to continue.

- The study doctor determines that you should discontinue your participation in the study, from the viewpoint of your safety (signs of illness, side effects and laboratory test results are also taken into consideration)
- If you are or become pregnant.
- If the company that requests the study or your attending investigator decides to discontinue the study
- If you wish to quit smoking during the baseline period, or during the exposure period after you are assigned to the Glo, commercially-available THP or conventional cigarette group.

In addition, we may discontinue your participation in the study in the following cases.

- If we lose contact with you (between screening and admission, or admission and follow up)
- If you take a drug without consultation with your study doctor (as a rule, for use of drugs, please consult your study doctor prior to use)
- If you don’t observe instructions of your attending investigator or the person in charge in the research site.

3.6 Discontinuation of the study

The Sponsor can request that the study is discontinued at any time. If the study is discontinued, we will inform you and take appropriate measures for your safety and the protection of your health.

1. **COURSE OF THE STUDY**
   1. **Screening**

This visit helps the Study Doctor determine if you qualify for this study. This is called the screening visit and involves the following procedures:

- Provide confirmation of your age (in the form of drivers’ license or passport) to enable the study doctor to verify your age. To enter this study you must be aged at least 23 years old.
- Read and sign this Informed Consent Form if you wish to participate
- Provide your medical history including your name, age, sex, race, and ethnicity
- An ECG - electrocardiogram (a test that measures and records the electrical activity of your heart) will be done
- A physical examination will be done
- Vital signs (pulse rate, blood pressures, body temperature and respiratory rate) and your height and body weight will be measured
- A Lung function test. At Screening this test will be performed with and without a bronchodilator. A bronchodilator is a type of medicine that makes breathing easier by relaxing the muscles in your lungs and widening the airways.
- An urine pregnancy test will be carried out in females
- Your urine will be screened for drugs of abuse and your breath tested for alcohol
- Your urine will be tested for cotinine (cotinine is a breakdown product of nicotine) and your breath will be tested for carbon monoxide. Both of these tests provide evidence that you are in fact a smoker.
- Nicotine Use Assessment – you will be asked to provide your tobacco and nicotine use history and to complete a questionnaire to assess your nicotine dependence. To enter this study you must have been a smoker for a minimum of three years, smoking at least 10 factory-made cigarettes up to a maximum of 30 per day with an ISO tar yield of 6-8mg for the previous six months, and are not planning on or trying to quit. Additionally, you must not be a current or recent (i.e. within 14 days prior to the screening visit) user of any other nicotine or tobacco product.
- Blood and urine samples will be collected. Your blood and urine sample will be used for routine laboratory tests. Your blood will also be used to test for HIV, hepatitis B and C and Syphilis. If your result for one of these tests shows positive, your sample will be sent to an external laboratory for confirmation that the result is correct as false positives sometimes occur i.e. the test shows a positive result but you may not be infected.
- You will be given a demonstration of the study THP products at Screening.
  1. **Admission**

You will be asked to arrive at the research site in the afternoon for check-in.

On arrival you will be required to give any cigarettes that you have brought with you to the research site staff. They will be returned to you when you leave the research site at the end of the study.

The following procedures will take place when you check-in, to ensure that no changes which may affect your safety and/or the study results have occurred since your screening visit:

- A sample of your urine will be collected and screened for drugs of abuse, and tested for cotinine
- If you are female, a urine pregnancy test will be performed
- An alcohol and carbon monoxide breath test will be performed
- A brief physical examination will be done
- Vital signs (pulse rate, blood pressures, body temperature and respiratory rate) and your body weight will be measured
- Any changes in your medical history, including any illnesses and/or use of any medication since your screening visit, will be discussed and documented
- Blood and urine samples will be collected for routine safety checks.
- A lung function test (with and without bronchodilator)
- After all admission tests are confirmed including the female pregnancy test, you will be asked if you want to try the study THP(s). You may try up to 3 THP sticks.

You will then be instructed on how to request your assigned product from the research site staff during the study, and how to return the used product to research site staff after use. From a time which will be confirmed by the research site staff, you will be free to request your assigned product as and when you wish. From the evening of Day -1, all of your urine will be collected from this point forwards.

On this day, you will be provided with dinner and a snack.

Designated smoking room will be closed at approximately 00:00.

- 1. **DAY 1 AND DAY 2**

On day 1 and day 2 you will be free to request the cigarette assigned to you (non-mentholated cigarette for study groups A, B, E and F, or mentholated cigarette for study groups C and D) as and when you wish to smoke. Your urine will continue to be collected throughout these days. At a specific time on both days you will be asked to refrain from smoking for at least 30 minutes before providing a carbon monoxide breath test.

On the afternoon of day 2, a sample of your blood will be collected

Depending on which study group you were assigned to, on the evening of day 2 you may be told that you must no longer smoke or use any tobacco products (smoking abstinence group F), or that you will stay on your assigned cigarette (study groups A and C) or now be permitted to use only the tobacco heating product assigned to you (study groups B, D and E), for the rest of your stay in the research site.

Product use will be restricted to between the hours of 06:00 and 00:00.

You will be provided with breakfast, lunch, dinner and a snack on both days.

- 1. **Evening of Day 2, and Days 3, 4, 5 and 6**

On the evening of day 2, and on days 3, 4, 5 and 6, you will be free to request your assigned product as and when you wish, unless you are in the group which has been instructed to stop using any tobacco products. Your urine will continue to be collected throughout. At a specific time on each day you will be asked to refrain from smoking for at least 30 minutes before providing a carbon monoxide breath test.

For practical reasons, product use will be restricted to between the hours of 06:00 and 00:00.

On the afternoon of day 5, a sample of your blood will be collected.

You will be provided with breakfast, lunch, dinner and a snack on each day.

- 1. **Day 7**

On day 7 you will be free to request your assigned product as and when you wish, unless you are in the group which has been instructed to stop using any tobacco products. Your urine will continue to be collected throughout. At a specific time on day 7 you will be asked to refrain from smoking for at least 30 minutes before you provide a carbon monoxide breath test.

On the afternoon of day 7, a sample of your blood will be collected.

You will be provided with breakfast, lunch and dinner on this day.

**If you are in the group which was instructed to stop using any tobacco products, you will be discharged from the research site on the evening of day 7.**

If you are in any of the other study groups, you will at a specific time on the evening of day 7 be instructed to stop using any tobacco products. At this point, collection of your urine will also stop.

- 1. **Day 8**

On the morning of day 8 you will be provided with a light breakfast or a snack.

At a specific time on the morning of day 8, you will be asked to smoke a single cigarette or use a single tobacco stick of your assigned product. The number of individual puffs you take will be recorded by the research site staff. A needle or catheter will be used to take blood samples from a vein in your arm a few minutes before, during, and for up to four hours after you use the product. You will not be permitted to use your assigned product again, or to use any other nicotine or tobacco products, during this period.

In total, about 13 samples totaling around 52 mL of your blood will be taken during this period. You will also be asked to complete a short questionnaire at a specific time after you use the product, to assess your satisfaction with that product.

After the last blood sample is taken you will complete discharge procedures before being released from the research site.

- 1. **Discharge**

Before you leave the research site, the study doctor will assess your health and well-being and will offer smoking cessation advice.

If you wish to continue smoking your own brand cigarettes after completion of this study you may smoke one cigarette before leaving the research site. This is because you may feel light-headed when you first smoke after the period of abstinence.

The following procedures will take place before you check-out of the research site, to ensure that no changes which may affect your safety and/or the study results have occurred since your screening visit and during your time on the study:

- If you are female, a urine pregnancy test will be performed
- A brief physical examination will be done
- Vital signs (pulse rate, blood pressures, body temperature and respiratory rate) and your body weight will be measured
- Follow up on any symptoms/signs of illness or side effects that you may have reported during the study.
- Blood and urine samples will be collected for routine safety checks.
- An ECG and lung function test with and without bronchodilator will be performed.
  1. **Follow-up telephone call**

The study staff will contact you by phone within 5 to 7 days after your discharge from the research site. You will be asked about any unusual symptoms/signs of illness or side effects you may have experienced since leaving the research site.

Following the end of the safety follow up period, you should not donate blood for 12 weeks (male) or 16 weeks (female).

- 1. **Early Termination**

If your participation in the study ends early and you do not complete all of the intended days within the study, then you will complete the discharge and follow-up procedures as described in sections 4.7 and 4.8 of this document when your participation in the study ends.

1. **PROCEDURES AND POSSIBLE RISKS OR DISCOMFORTS**

Procedures will be done during the study at assigned times. You will be given a schedule of all study procedures. The procedures will be done to monitor aspects of your health, assess your use of the study product, and to see how the study product is broken down in your body. In the instance that the study doctor detects any abnormalities in your health, based on the results of study procedures such as ECG, physical examinations or safety blood draw results, they will discuss this with you immediately. If necessary they might contact your GP directly or provide you with a letter to take to your GP.

- 1. **TEST PRODUCTS**

Tobacco products are addictive and their consumption is associated with real risks of serious diseases. The best way to avoid the risks associated with tobacco products is not to use them at all.

Nicotine and tobacco use can have side effects but as you are already using tobacco products the risks related to the side effects of nicotine through study product administration are low. During study product use, you are not likely to be exposed to nicotine levels higher than the ones you are usually exposed to during your daily consumption of tobacco products.

The most common short-term side effects related to the use of tobacco products include: cough, irritation in the mouth and throat, palpitations, feeling faint, nausea, dizziness and headache.

Less common side effects include: nasal congestion, stomach discomfort, hiccups and vomiting.

Even less common are: cardiac arrhythmia.

There is always a chance that an unexpected or serious side effect may happen. You must report any new symptoms/signs of illness to the study doctor or person in charge any time after you have signed this informed consent form.

When using any of the THPs (Glo or commercially-available THP), you will be provided with a fully charged device. If the device appears to fail, overheat, or malfunction in any way, please immediately stop using the device and report this to the person in charge.

Tobacco consumables should only be used with the Glo and commercially-available THP devices, respectively. You must not attempt to light or smoke the tobacco consumables in any other way.

- - 1. **Risks to an unborn baby or child who is breastfeeding:**

The risks of using nicotine containing products during pregnancy are not known. It is possible that they may cause harm to an unborn baby. This may include death, congenital malformations or other unforeseen health problems for the baby.

It is recommended that you avoid sexual contact during the study to avoid pregnancy. If you choose to be sexually active during the study, you must use an acceptable method of birth control. The study staff will discuss with you what an acceptable method of birth control is. A pregnancy may still occur even while using birth control. Not having sex with a person of the opposite sex is the only way to be certain that a pregnancy will not occur.

- - - 1. **Females**

If you are able to become pregnant, you must use an approved method of birth control during the entire period between your screening visit and the end of the study.

The study is considered to be complete after the safety follow up period (once you have received the final follow up phone call from the study coordinator)

The potential risks of the study product to a baby during breastfeeding are not known. Therefore women who are nursing cannot take part in this study.

- - - 1. **Males**

You must use an approved method of birth control during the entire study. You must not donate sperm during this time. The study is considered to be complete after the safety follow up period (once you have received the final follow up phone call from the study coordinator)

- - 1. **Smoking cessation**

If you are assigned to the group which is not permitted to use any tobacco product after visit day 2 you may experience potential effects of tobacco use abstinence, e.g. irritability, anxiety, nausea, cravings for tobacco etc.

- 1. **Blood Collections**

If you are assigned to study group A, B, C, D or E a needle or catheter will be used to take blood samples from a vein in your arm about 22 times during the course of the study. About 100 mL of blood will be taken during the course of the whole study.

If you are assigned to group F (smoking abstinence) then a needle or catheter will be used to take blood samples from a vein in your arm about 9 times during the study. About 48 mL of blood will be taken during the study.

Additional samples may need to be taken for example if we need more information due to you experiencing a side-effect. No more than 150 mL of blood will be taken during the study.

An IV catheter may be inserted into a vein in your hand or arm. Blood is then withdrawn from a port on the catheter at scheduled time points. The tube may be flushed or cleaned out with a small amount of saline (salt water) before and after it is used, this is to help keep blood flowing through the catheter. You may have discomfort or pain when the catheter is inserted. There is a risk of infection, bleeding and/or bruising at the insertion site.

You may have discomfort or pain when your blood is collected. You may feel faint or pass out (or faint). There is a risk of infection, bleeding, or bruising at the puncture site. You may develop a small scar at the puncture site where multiple blood samples are taken.

- 1. **Urine samples and collections**

In this study, spot urine (urine samples collected at specific times) will be used to perform the safety lab analysis, urine drug test, urine cotinine test and urine pregnancy test for females.

During the study, 24 hour urine collections will be performed. For the 24 hour urine collection you will need to collect all of your urine into a collection bottle. The study coordinator in charge will provide you with a bottle when you need to urinate. Further details will be explained by the study coordinator in charge.

1. **Persons who can participate in this study**

Once you have understood the content of the study and given your consent to participate, the Investigator will review a further set of requirements, called inclusion and exclusion criteria, to confirm that you are appropriate to take part in the study. The following list contains the main criteria. For more details, please ask for the study Investigator to explain.

**Inclusion Criteria**

- A person who has signed the informed consent form and fully understands the requirements of the study.
- 23 years or older, but not older than 55
- Japanese
- A person who is determined by the study doctor to be healthy, based on results of the medical history, physical examination, safety laboratory test, ECG, lung function test, vital signs (pulse rate, blood pressure, body temperature and respiratory rate) and weight assessment
- Current smokers, who, based on self-report, have been smokers continuously for 3 years or more, and smoke between 10 and 30 conventional cigarettes per day.
- Smoked the same brand for a minimum of 6 months and whose chosen brand contains between 6 mg/cig and 8 mg/cig tar (as determined under ISO smoking regime).
- A person who can accept using the study test products, either Glo and Neostik, commercially-available THP with tobacco consumable, or the study conventional cigarette
- A person who can accept a period of smoking abstinence for 5 days maximum if assigned to the smoking cessation group.

**Exclusion Criteria**

- The Investigator may decide, based on your medical history, that you are not suitable for the study.
- The Investigator may decide, based on your current medical results at screening, that you are not suitable for the study.
- Female subjects who are pregnant or breastfeeding or who do not agree to use a highly effective method of birth control from the time of signing the consent form to the end of the follow up visit.
- Male subjects who do not agree to using an effective form of contraception in addition to a highly effective second form of contraception used by their female partners from the time of signing consent form to the end of the follow up visit.
- Male subjects who intend to donate sperm between admission and the end of the follow up period.
- Male subjects who have donated blood (≥400 mL) within 12 weeks prior to admission, plasma within 2 weeks of admission or platelets within 6 weeks of admission.
- Female subjects who have donated blood (≥400 mL) within 16 weeks prior to admission, plasma within 2 weeks of admission or platelets within 6 weeks of admission.
- If you have used a nicotine containing product other than conventional cigarettes such as nicotine gum an electronic tobacco product, or similar device, within 14 days of screening.
- A positive drug or alcohol test.
- A positive test for Human Immunodeficiency Virus (HIV),Hepatitis B/C or Syphilis
- If you have used some types of medical drugs (prescribed or commercially available drugs) within 14 days before screening or admission to the study.
- Employees, or their relatives, who are working now or who have worked for a tobacco company.
- Employees, or their relatives of the investigation research site.
- Persons who are not able to fully understand the consent form.
- Persons who have previously been enrolled or withdrawn from this study.
- Persons who have previously been diagnosed with any form of malignancy

Based on the results obtained at the Screening visit and with consideration of the Inclusion and Exclusion criteria, the Investigator will decide whether you can participate in the study or not.

1. **SIGNIFICANT NEW SAFETY FINDINGS DURING THE STUDY**

You will be told of any significant new safety findings that the Investigator is made aware of by the Sponsor that might influence your willingness to continue your participation in this study.

1. **POSSIBLE BENEFITS FROM THE STUDY**

You will not receive any health benefits from being in this study. The tests provided may help you learn about your general health. They may also help you discover an unknown medical condition. This study may help doctors, scientists or manufacturers learn things about tobacco products that will help others to quit smoking.

You can speak to the study doctor at any time to ask for advice on completely stopping using tobacco products.

1. **WHAT WILL HAPPEN TO ANY SAMPLES I GIVE?**

The samples of your blood taken at the screening visit, on the afternoon of research site check-in and at discharge will be shipped to LSI Medience Corporation, Tokyo, Japan and tested for laboratory safety parameters. They will also test the samples of your blood taken at the screening visit for HIV, viral hepatitis and syphilis, and (for female subjects) pregnancy.

The samples of your urine taken at the screening, research site check in and discharge visit will be shipped to LSI Medience Corporation, Tokyo, Japan and tested for laboratory safety parameters.

The samples of your urine taken at screening and research site check in will be tested at site for cotinine, drugs of abuse and microscopy.

For female subjects, the samples of your urine taken at research site check in and discharge will also be tested for pregnancy.

The samples of your urine collected from the evening of research site check-in to the evening of day 7 will be shipped to ABF GmbH, Munich, Germany and Celerion Inc., Lincoln, NE, USA for biomarker analysis and may be kept for up to *one year* after testing, after which they will be destroyed.

The samples of your blood taken on days 2, 5 and 7 will be shipped to LSI Medience Corporation, Tokyo, Japan for biomarker analysis, and may be kept for up to *one year* after testing, after which they will be destroyed.

The samples of your blood taken on day 8 will be shipped to Celerion Inc., Lincoln, NE, USA for nicotine analysis, and may be kept for up to one year after testing, after which they will be destroyed.

If the results from testing your blood or urine samples indicate there may be something wrong with your health, we will discuss this with you at the time. You will not be told of the results of the analyses on your samples for levels of biomarkers, nicotine, metabolites or nicotine effect.

- **WHAT SAMPLES WILL BE USED FOR:**

Your samples will be used for the research purposes explained in this ICF.

Your samples will not be sold or used directly for the production of commercial products.

In case of any commercial gain based on research results from your samples, the sponsor will have the ownership of the research results and may file patents. The research done with your samples may help to develop new products, new medical tests or treatments in the future that have commercial value. There will be no financial benefit to you for any commercial findings or products as a result of your sample use. By agreeing to take part in this research study, you agree to give up your rights for any commercial value resulting from your samples and data.

Your samples will be provided to third party laboratories for testing, research use and storage purposes done for and on behalf of the sponsor of this study and its third party collaborators

Your samples will be coded to protect your identity.

Samples will be identified with a unique code; this will not include your initials or date of birth unless these are considered medically relevant. Date of birth will only be collected if medically relevant to this study, unless it is legally restricted in applicable jurisdictions of collection and/or storage.

Reports about research done with your samples will not include any details about or be put in your health/medical record and will be kept confidential to the best of our ability within the law.

In the future, researchers studying your samples may need to know more about you, such as whether you smoke or not, and other information such as your age, gender, race. If this information is already available because you are taking part in a study, it may be given to the researcher but it will not contain information that might reveal your identity.

1. **TAKING PART IN THE STUDY OF YOUR OWN FREE WILL**

You are being asked to take part in this study because you are healthy. You will not be taking the study products to treat any disease or condition. The only other option is not to take part in this study.

If you choose to take part in the study you will do so by your own choice and your own free will. No one can force you to be in the study. If you enter the study, no one can force you to stay in the study. If you choose not to be in the study or if you leave the study early, there will be no penalty. If you leave or are removed from the study for any reason your financial compensation will be prorated to the amount of the study that you complete. You will not lose any rights that you are entitled to as a research subject.

1. **COST AND PAYMENT FOR TAKING PART IN THE STUDY**

There are no costs to you for being in the study. The study products and study procedures are provided to you at no charge.

*The research site* will pay you by *<insert payment method>* on completion of the study.

The amount you will be paid will depend on how much of the study you complete:

*<Insert payment schedule>*

You will be paid a total of <xxxx> Japanese Yen. You will earn only *<insert standby stipend>* if you are chosen as a standby and never enter the study exposure period.

If you leave the study early, you will receive a pro-rated amount based on the study days you completed.

You understand the following:

- No deductions will be withheld from your stipend for tax purposes.
- You are responsible for reporting any payment on your individual tax returns and for payment of any applicable taxes.
- Being in this study does not make you an employee of the Sponsor, research site or any third party vendors.
- You will not receive the full payment for the study if you leave before it is complete.
  **This may include leaving the study due to a symptom/sign of illness or side effects.**

1. **COMPENSATION FOR AN INJURY DIRECTLY RELATED TO YOUR PARTICIPATION IN THIS STUDY**

There is a chance that you could become ill or injured while being in this study. If your illness or injury relates to your participation in the study and requires medical care outside of the research site *(i.e. hospital, ambulance, medical specialist),* the research site will help to make arrangements for this care.

- 1. **What happens if the injury or illness is a direct result of the study?**

The Study Doctor will decide if an injury or illness is directly related to the performance of the protocol (study plan) or use of the study products. If your injury or illness is directly related to the performance of the protocol (study plan) or use of the study products the Sponsor and/or the research site will ensure you receive the appropriate treatment either within the national healthcare sector or, if appropriate, a private healthcare provider. The Sponsor holds appropriate clinical trials and other applicable insurance to cover the conduct of this study.

You will need to sign a “release of information” form in this event. This form will allow the research site to obtain your medical records related to the illness or injury. These records will help the study doctor determine the cause of the illness or injury. They may also help the Sponsor learn more about the safety of the study product.

1. **REASONS YOU CAN BE REMOVED FROM THE STUDY**

**You can be removed from the study at any time and for any reason without your consent.** Some of the reasons you can be removed are listed below:

- You do not follow the instructions, rules, and restrictions given by the study staff.
- You do not continue to meet the requirements for the study.
- The Study Doctor decides it is best for your health.
- The Sponsor stops the study or asks that you be removed from the study.
- A business or commercial decision in the best interests of the sponsor
- Decisions made by the Institutional Review Board
- You become pregnant.

1. **LEAVING THE STUDY BEFORE IT IS COMPLETED**

If you choose to leave the study, you must notify the study doctor or study staff. You will be asked to complete the discharge procedures prior to leaving the research site. You will then enter the safety follow up period of between 5 and 7 days.

- You will be telephoned and asked about any side effects or medications you may have taken.

All data and human biological samples that have been collected prior to you leaving the study will be used for purposes specified in the ICF.

1. **CONFIDENTIALITY**

Information about you and your participation in this study will be kept confidential according to privacy laws in Japan. Your original study records may contain your name and other personally identifiable information (PII). PII is information that directly identifies you. These records and your study results will be kept by the research site on paper and/or in a database as required by law or the Sponsor. This may be indefinitely. Your study results will be coded with numbers. The research site will keep a list that links your name to your study results. This list will be kept confidential.

The study results may be audited and monitored by the people listed below. This is to make sure the study was done correctly. In order for this to take place, some third parties will have direct access to and may copy some of the original records. This includes the laboratory report linking your name to your HIV and/or hepatitis and/or syphilis test results. Your original records may contain your PII. These third parties include:

- Regulatory authorities, such as the Japan Pharmaceuticals and Medical Devices Agency, United States Food and Drug Administration, UK Medicine and Healthcare products Regulatory Agency, European Medicines Agency or Health Canada
- The Sponsor and third parties working with the Sponsor
- The research site and third parties working with the research site.
- The Institutional review board (IRB) – this is a group of people who review research studies to protect the rights and welfare of research participants.

All of the parties listed above will maintain, use, disclose, transfer and access your PII confidentially. This may happen anywhere in the world. This is done in accordance with applicable law or regulation.

If any or part of the study test results are published, it will be published in a way to maintain your confidentiality.

If a medical emergency happens, your study results may be given to emergency medical staff not employed by the research site, or the Sponsor. If you decide to stop being in the study, the information already gathered will still be kept in the study database. It will be used as described in this Informed Consent Form. In accordance with applicable law, you may request (in writing) to see or have a copy of the study data collected about you. You can also request to correct any information that is not correct. You may not be able to see some data until after the study is over.

By signing this form, you are allowing the use and disclosure of your personal data as described in this informed consent form.

1. **WHO WILL ANSWER YOUR QUESTIONS?**

You can ask questions about this consent form or the study (before you decide to start the study or at any time during the study). Questions may include:

- Who to contact in the case of a research-related injury or illness
- Any payment for being in the study
- Your rights and your responsibilities as a study subject
- Other questions

Contact the Study Doctor or study staff with any questions or concerns.

The names and telephone numbers of the study staff to contact are listed in the table below.

| **Principal Investigator** | <NAME> | <CONTACT NUMBER> |
| --- | --- | --- |
| **Study coordinator** | <NAME> | <CONTACT NUMBER > |

1. **ACKNOWLEDGEMENT**

You have been given a signed copy of this document. Subject’s initials ________

- 1. **SUBJECT RESPONSIBILITIES**

You must:

- Inform us of any medical condition you have been diagnosed with.
- Follow all research site rules and instructions of the study staff.
- Follow the study restrictions.
- Report any new symptoms/signs of illness or side effects.
- Report any failure, overheating or other malfunction of the study products.
- Give true and complete answers to any questions.
- Comply with the terms of the Informed Consent Form.
  1. **GENERAL RESEARCH SITE RULES AND STUDY RESTRICTIONS**

Some drugs, foods, drinks, or activities can increase or decrease the effect of the study product. This can be a risk to your health or lead to false study results. The restrictions for this study are listed below:

- You should avoid unusually intense or strenuous exercise from 7 days prior to your screening visit and throughout the study
- You should not eat food containing poppy seeds for 3 days prior to your screening visit, and for 3 days prior to research site check-in
- You should not drink alcohol for 3 days prior to your screening visit, and for 3 days prior to research site check-in
- You should not eat cruciferous vegetables e.g. wasabi, horseradish, broccoli, cabbage, radish, Brussels sprouts, Watercress, Rapeseed oil and Canola for at least 2 days prior to research site check-in
- You should not eat any grilled, smoked, fried or barbequed food during the 2 days prior to research site check-in, nor should you be present during the cooking of such food.
- You should not use any prescription, over the counter medications or herbal medications (with the exception of hormonal contraceptives) from the screening visit and throughout the study
- You must not smoke any tobacco or use any nicotine products, other than those assigned to you, from check-in to the research site until discharge from the research site at the end of the study
- You should not donate blood for at least 12 weeks if you are male and 16 weeks if you are female after the end of safety follow up period. You must also inform research site staff if you have donated blood in the last 3 months.

1. **INFORMED CONSENT FORM**

I hereby confirm that:

- I have been informed by Study Doctor about the purposes, type and character of the medical experiment and about the risks and benefits related to participation in this study.
- I have read and understood the information in this written Informed Consent Form.
- A study staff member has explained this form to me.
- I have had the chance to ask questions about the study and I have received satisfactory answers to all of them.
- I agree to follow the restrictions of the study.
- I agree to take part in the study and voluntary give my consent for study procedures.
- I agree for my blood and urine samples to be collected for laboratory and biomarker analysis.
- I agree for my blood samples to be collected and analysed in order to rule out infection with HIV virus, Hepatitis C virus, Hepatitis B and Syphilis.
- I am aware that nothing contained in this informed consent form waives any of my legal rights as a research subject, nor does it release the Study Doctor, the Sponsor, the research site, third party vendors, or its agents from any liability for negligence.
- I have been informed about the third party insurance of the Sponsor for damages incurred as a result of participation in this study, and I accept them.
- I understand that I am free to withdraw from the research at any time without having to give a reason.
- I confirm I am a regular smoker of between 10 and 30 cigarettes per day, and do not plan to quit smoking in the next 12 months.
- I confirm I am over the age of 23 years.
- I confirm that I am not pregnant or nursing/breastfeeding.
- I agree to keep confidential all information relating to the study product, including the product design, specifications and method of operation.
- I understand that the Sponsor, the Sponsor’s designees, the monitor(s), the auditor(s) and the Institutional Review Board (IRB) will be granted direct access to my medical records for verification of study procedures and/or data without violating my confidentiality.
- I agree that my general practitioner (GP) will be informed of my participation in this study if required.

Please provide the following details:

Name and Address of GP: ____________________________________________________________________

Subject Name (Print): ____________________________________________________________________

Subject Signature: ____________________________________________________________________

Date of subject signature: _______________________ Time of subject signature: _______________________

1. **FOR RESEARCH SITE INVESTIGATOR**

I have discussed this study with the above subject. This person had an opportunity to ask questions. The Subject signed this ICF in my presence.

Investigator obtaining the Informed Consent of Subject:

Investigator Name (Print): ___________________________________________________________

Investigator Signature:______________________________________________________________

Investigator Signature Date and Time: __________________________________________________
