# Supplementary material for: A randomised, controlled, two-Centre open-label study in healthy Japanese subjects to evaluate the effect on biomarkers of exposure of switching from a conventional cigarette to a tobacco heating product
Source: BMC Public Health. 2017 Aug 22;17:673. doi: 10.1186/s12889-017-4678-9 (PMC5567437; doi:10.1186/s12889-017-4678-9)
Supplement: Supplementary file 2 — Appendix 2: Statistical Analysis Plan. (DOCX 96 kb) [file 12889_2017_4678_MOESM2_ESM.docx]

**Appendix 2.**

**Statistical Analysis Plan.**

STATISTICAL ANALYSIS PLAN

**A Randomised, Controlled, Multi-centre Open-label Study in
Healthy Japanese Subjects to Evaluate the Effect on Biomarkers of
Exposure of Switching from a Conventional Combustible Cigarette
to the Glo Tobacco Heating Product**

Statistical Analysis Plan Status: Final v2
Statistical Analysis Plan Date: 29 June 2017

Study Products: Lucky Strike Regular Combustible Tobacco Cigarette, Glo Device with Neostik, Lucky Strike Menthol Combustible Tobacco Cigarette, Glo Device with Mentholated Neostik, and iQOS Device with Marlboro HeatStick (Regular).

Sponsor Reference Number: BAT3416008
Covance Study Number: 8355511

|  |  |
| --- | --- |
| Sponsor:  British American Tobacco (Investments) Ltd.  R&D Centre Regents Park Road  Southampton  Hampshire  SO15 8TL, UK | Study Sites: Fukuoka Mirai Hospital, Fukuoka  and  Hakata Clinic, Fukuoka |
|  |  |
| Sponsor Signatory: Graham Errington, BSc, MBA | Principal Investigators: Tatsuya Yoshihara and Miwa Haranaka |
|  |  |

1. Statistical analysis plan Approval Signatures

By signing this page when the Statistical Analysis Plan (SAP) is considered final, the signatories agree to the statistical, pharmacokinetic (PK), and biomarker analyses to be performed for this study, and to the basic format of the tables, figures, and listings (TFLs). Once the SAP has been signed, programming of the TFLs based upon this document can proceed. Any modifications to the SAP and TFLs made after signing may result in a work-scope change.

**Covance approval:**

|  |  |  |
| --- | --- | --- |
| Andrew Hedge, BSC, MSc  **Statistician** |  | **Date** |
|  |  |  |
| Stuart Hossack, BSc  **Pharmacokineticist** |  | **Date** |

**Sponsor approval:**

|  |  |  |
| --- | --- | --- |
| Graham Errington, BSc, MBA  **Senior Statistician** |  | **Date** |

1. Table of Contents

[1 Statistical analysis plan Approval Signatures 2](#_Toc486511753)

[2 Table of Contents 3](#_Toc486511754)

[3 ABBREVIATIONS 5](#_Toc486511755)

[4 VERSION HISTORY 7](#_Toc486511756)

[5 Introduction 7](#_Toc486511757)

[6 STUDY OBJECTIVES and Endpoints 8](#_Toc486511758)

[7 STUDY DESIGN 9](#_Toc486511759)

[8 PRODUCTS 11](#_Toc486511760)

[9 SAMPLE SIZE JUSTIFICATION 11](#_Toc486511761)

[10 Definition of Analysis Populations 11](#_Toc486511762)

[11 Statistical Methodology 12](#_Toc486511763)

[11.1 General 12](#_Toc486511764)

[11.1.1 Definition of Baseline and Change from Baseline 12](#_Toc486511765)

[11.1.2 Repeat and Unscheduled Readings 13](#_Toc486511766)

[11.2 Demographics and Subject Disposition 13](#_Toc486511767)

[11.2.1 Baseline Smoking Questionnaire 13](#_Toc486511768)

[11.2.2 Medical History 14](#_Toc486511769)

[11.3 Pharmacokinetic Assessment 15](#_Toc486511770)

[11.3.1 Pharmacokinetic Analysis 15](#_Toc486511771)

[11.3.2 Criteria for handling concentrations below the limit of quantification in Pharmacokinetic analysis 15](#_Toc486511772)

[11.3.3 Calculation of AUC 16](#_Toc486511773)

[11.3.4 Anomalous Values 16](#_Toc486511774)

[11.4 Presentation of Pharmacokinetic Data 16](#_Toc486511775)

[11.4.1 Presentation of Pharmacokinetic Plasma Drug Concentration Data 16](#_Toc486511776)

[11.4.2 Presentation Pharmacokinetic Parameters 16](#_Toc486511777)

[11.5 Biomarkers of Exposure 17](#_Toc486511778)

[11.5.1 Biomarker of Exposure Analysis Variables 17](#_Toc486511779)

[11.5.2 Biomarker of Exposure Statistical Methodology 19](#_Toc486511780)

[11.6 Biomarkers of Biological Effect 20](#_Toc486511781)

[11.6.1 Biomarker of Biological Effect Analysis Variables 20](#_Toc486511782)

[11.6.2 Biomarker of Biological Effect Statistical Methodology 20](#_Toc486511783)

[11.7 Safety and Tolerability Assessments 20](#_Toc486511784)

[11.7.1 Adverse Events 20](#_Toc486511785)

[11.7.2 Clinical Laboratory Parameters 21](#_Toc486511786)

[11.7.3 Vital Signs 21](#_Toc486511787)

[11.7.4 Electrocardiogram 21](#_Toc486511788)

[11.7.5 Lung Function Tests 21](#_Toc486511789)

[11.7.6 Physical Examination 21](#_Toc486511790)

[11.7.7 Other Safety Assessments 22](#_Toc486511791)

[11.8 Other Assessments 22](#_Toc486511792)

[11.8.1 Product Satisfaction 22](#_Toc486511793)

[11.8.2 Product Use 22](#_Toc486511794)

[11.8.3 Puff Count 22](#_Toc486511795)

[12 INTERIM analyses 22](#_Toc486511796)

[13 changes from the protocol specified statistical analyses 23](#_Toc486511797)

[14 DAta Presentation 23](#_Toc486511798)

[14.1 Insufficient Data for Presentation 23](#_Toc486511799)

[15 References 23](#_Toc486511800)

1. ABBREVIATIONS

Abbreviations pertain to the SAP only (not the TFLs).

| 3-HPMA | 3-hydroxypropylmercapturic acid |
| --- | --- |
| AAMA | N-acetyl-S-(2-carbamoylethyl)cysteine |
| ADaM | analysis data model |
| Ae | amount excreted |
| AE | adverse event |
| ANOVA | analysis of variance |
| AUC | area under the concentration time curve |
| AUC_0-last_ | area under the concentration-time curve from time zero to the time of the last quantifiable concentration |
| BLQ | below the limit of quantification |
| BoE | biomarkers of exposure |
| BoBE | biomarkers of biological effect |
| CDISC | Clinical Data Interchange Standards Consortium |
| CEMA | 2-cyanoethylmercapturic acid |
| CI | confidence interval |
| C_max_ | maximum observed plasma concentration |
| CO | carbon monoxide |
| CSR | Clinical Study Report |
| CV | coefficient of variation |
| EC | Early Clinical |
| ECG | electrocardiogram |
| FEF_25-75%_ | Forced Expiratory Flow 25-75% |
| FEV_1_ | Forced Expiratory Volume after 1 second |
| FVC | Forced Vital Capacity |
| HEMA | 2-hydroxyethylmercapturic acid |
| HMPMA | 3-hydroxy-1-methylpropylmercapturic acid |
| ICH | International Conference on Harmonisation |
| ISO | International Organisation for Standardisation |
| ITT | intent-to-treat |
| LS | least squares |
| MedDRA | Medical Dictionary for Regulatory Activities |
| MHBMA | Monohydroxybutenyl-mercapturic acid |
| NGP | next generation product |
| NC | not calculated |
| o-Tol | o-Toluidine |
| PEAE | product-emergent adverse event |
| PK | pharmacokinetic |
| PP | per protocol |
| QTc | QT correction; QT interval corrected for heart rate |
| QTcB | QTc calculated using the Bazett correction |
| QTcF | QTc calculated using the Fridericia correction |
| S-PMA | S-phenylmercapturic acid |
| SAE | serious adverse event |
| SAP | Statistical Analysis Plan |
| SD | standard deviation |
| TFLs | tables, figures, and listings |
| THP | tobacco heating products |
| TNeq | Total Nicotine Equivalents (nicotine, cotinine, 3-hydroxycotinine and their glucuronide conjugates) |
| t_max_ | time of maximum observed concentration |

1. VERSION HISTORY

| Version | Date | Amendments |
| --- | --- | --- |
| Final | 23 February 2017 | None - original document |
| Final v2 | 29 June 2017 | - Amend error in formula for calculation of TNeq - Add units for amount excreted parameters - Added rules for handling <LOD |

1. Introduction

This SAP has been developed after review of the clinical study protocol (Final Version 3.0 dated 25 January 2017).

This SAP describes the planned analysis of the safety, tolerability, PK, and biomarker data from this study. A detailed description of the planned TFLs to be presented in the Clinical Study Report (CSR) is provided in the accompanying TFL shell document.

The intent of this document is to provide guidance for the statistical analyses of PK and biomarker data. In general, the analyses are based on information from the protocol, unless they have been modified by agreement between British American Tobacco (Investments) Ltd and Covance Early Clinical (EC) Biometrics. A limited amount of information concerning this study (e.g., objectives, study design) is given to help the reader’s interpretation. This SAP must be finalised prior to the lock of the clinical database for this study. When the SAP and TFL shells are agreed upon and finalized, they will serve as the template for this study’s CSR.

This SAP supersedes the statistical considerations identified in the protocol; where considerations are substantially different, they will be so identified. If additional analyses are required to supplement the planned analyses described in this SAP, they may be performed and will be identified in the CSR. Any substantial deviations from this SAP will be agreed upon between British American Tobacco (Investments) Ltd and Covance EC Biometrics and identified in the CSR.

This SAP is written with consideration of the recommendations outlined in the International Conference on Harmonisation (ICH) E9 guideline entitled, “Guidance for Industry: Statistical Principles for Clinical Trials” and the ICH E3 guideline entitled, “Guidance for Industry: Structure and Content of Clinical Study Reports.”**^Error! Reference source not found.^**^,^**^Error! Reference source not found.^**

1. STUDY OBJECTIVES and Endpoints

**Primary Objective**

The primary objective is:

To quantitatively assess within-arm changes in biomarkers of exposure (BoE) and biomarkers of biological effect (BoBE) (see below) following a forced switch from a conventional cigarette to a next generation product (NGP) or cessation.

**Secondary Objectives**

The secondary objectives are:

- To assess differences between arms in BoE and BoBE (see below) following a forced switch from a conventional cigarette to an NGP or cessation.
- To determine nicotine PK parameters for the study products.
- To assess subjects’ satisfaction with the study products.
- To monitor the safety profile of subjects using tobacco heating products (THPs) and conventional cigarettes, and subjects undergoing smoking cessation.

**Endpoints**

The BoE on this study are:

- Carbon monoxide (CO)
- Total Nicotine Equivalents (nicotine, cotinine, 3-hydroxycotinine and their glucuronide conjugates) (TNeq)
- Total 4-(methylnitrosamino)-1-(3-pyridyl)-1-butanol (Total NNAL)
- Total N-nitrosonornicotine (Total NNN)
- 3-hydroxypropylmercapturic acid (3-HPMA)
- 3-hydroxy-1-methylpropylmercapturic acid (HMPMA)
- S-phenylmercapturic acid (S-PMA)
- Monohydroxybutenyl-mercapturic acid (MHBMA)
- 2-cyanoethylmercapturic acid (CEMA)
- 4-aminobiphenyl (4-ABP)
- o-toluidine (o-Tol)
- 2-aminonaphthalene (2-AN)
- 1-hydroxypyrene (1-OHP)
- 2-hydroxyethylmercapturic acid (HEMA)
- N-acetyl-S-(2-carbamoylethyl)cysteine and N-acetyl-S-(2-hydroxy-2-carbamoylethyl) cysteine (AAMA and GAMA)

The BoBE on this study are:

- 8-Epi-Prostaglandin F2α Type III (8-Epi-PGF2α Type III)
- Total White blood cell (WBC) count

The PK endpoints are:

- Time to maximum observed plasma concentration (t_max_) for nicotine.
- Maximum observed plasma concentration (C_max_) for nicotine.
- Area under the nicotine plasma concentration-time curve from time zero to the last measurable concentration (AUC_0-last_).

The safety endpoints are:

- Medical history of study subjects.
- Physical examination.
- Vital signs.
- Electrocardiogram (ECG).
- Clinical laboratory evaluations.
- Lung function test.
- Adverse events (AE)/serious adverse events (SAE) recording.

Other endpoints are:

- Product satisfaction questionnaire.
- Puff count during PK Assessment Period.
- Creatinine concentration (24-hour urine).
- Product use during exposure phase.
- Nicotine molar metabolic ratio (3-hydroxycotinine / cotinine) in urine.

1. STUDY DESIGN

This is a multi-centre, controlled study utilising a randomised switching design for cigarette smoking, NGP use and cessation arms. A total of 180 healthy adult male and female regular smokers of 6-8 mg International Organisation for Standardisation (ISO) tar yield cigarettes will be recruited. This study will include 3 THP products (2 non-menthol and 1 menthol) and 2 conventional cigarette products (1 non-menthol and 1 menthol). The aim is for 30 subjects in every arm to complete the study. A schematic of the study design is shown below:

Subjects will reside at the clinical site from Admission on the afternoon of Day -1 until Discharge on Day 7 (Arm F) or after completion of the PK assessments on Day 8 (Arms A-E). A poststudy Follow-up assessment will be conducted via telephone 5-7 days after Discharge.

During the study smokers whose current own-brand is a non-menthol cigarette will only use non‑menthol product, whilst smokers whose current own-brand is a menthol cigarette will only use menthol product. Based on whether the subject’s own-brand is a menthol or non-menthol cigarette, subjects will be randomised to one of the following study arms:

|  | **Study Arm** | **Baseline Period** | **Exposure Period** | **PK Assessment Period** |
| --- | --- | --- | --- | --- |
|  |  | **Days -1 to 2** | **Days 2 to 7** | **Day 7-8** |
| **Non-Menthol** | **A** | Conventional cigarette  N = 120 | Conventional cigarette  N = 30 | Conventional cigarette  N = 30 |
|  | **B** |  | Glo THP with Neostik  N = 30 | Glo THP with Neostik  N = 30 |
|  | **E** |  | iQOS THP with HeatStick  N = 30 | iQOS THP with HeatStick  N = 30 |
|  | **F** |  | Smoking abstinence  (Cessation)  N = 30 | N/A |
| **Menthol** | **C** | Conventional mentholated cigarette  N = 60 | Conventional mentholated cigarette  N = 30 | Conventional mentholated cigarette  N = 30 |
|  | **D** |  | Glo THP with mentholated Neostik  N = 30 | Glo THP with mentholated Neostik  N = 30 |

Throughout the study subjects will only use the products assigned to them. During the Baseline and Exposure Periods, smoking will be *ad libitum*, subject to a maximum limit of 120% of their usual daily cigarette consumption based on their tobacco-use history recorded at Screening. The PK assessment will require a controlled, single session of product use. Subjects will be reminded of the dangers of smoking prior to enrolment onto the study and that they are free to voluntarily quit smoking and/or withdraw from the study at any time.

In all arms, subjects will smoke conventional cigarettes (non-menthol [Arms A, B, E and F] or menthol [Arms C and D]) during the Baseline Period (Day -1 to the evening of Day 2).

Based on study arm, subjects will continue using conventional cigarettes (Arms A and C), switch to an assigned THP (Arms B, D and E), or abstain from smoking (Arm F), until the end of the Exposure Period (Day 7).

1. PRODUCTS

The following is a list of the study product abbreviations and ordering that will be used in the TFLs.

| **Study Product Name** | **Abbreviation to be used in TFLs** | **Product Order on TFLs** |
| --- | --- | --- |
| Lucky Strike Regular Combustible Tobacco Cigarette | LS Regular | 1 |
| Glo Device with Neostik | Glo THP | 2 |
| Lucky Strike Menthol Combustible Tobacco Cigarette | LS Menthol | 3 |
| Glo Device with Mentholated Neostik | Glo THP Menthol | 4 |
| iQOS Device with Marlboro HeatStick (Regular). | iQOS THP | 5 |
| Smoking cessation | Cessation | 6 |

1. SAMPLE SIZE JUSTIFICATION

A sample size of 30 subjects per arm has been set for this study. This is based on powering the primary objective of within-arm comparison of biomarker levels at baseline and end-of-study. The calculation was based on the number of pairs required to perform a paired t-test with 80% power for a decrease in biomarker levels of 40% or more compared to historical biomarker data available for a 7 mg ISO tar conventional cigarette. A sample size of 30 was determined to be adequate based on the biomarker requiring the most pairs to power (exhaled CO) and allowing for attrition. A sample size of 30 should also provide sufficient power for the secondary objective of between-arm comparisons, based on a minimum of 40% reduction in BoE.

1. Definition of Analysis Populations

The **Safety Population** - All subjects who smoked at least one cigarette or had at least one safety assessment after enrolment on Day -1.

**Intent-to-treat population (ITT)** - All subjects who were assigned to a product and had at least one valid assessment of a biomarker variable.

**Per-protocol population (PP)** - All subjects who had valid assessment of a biomarker variable and completed study according to the protocol (no major protocol deviations).

The **PK Population** - All subjects who had sufficient data to calculate at least 1 PK parameter and completed the study up to the end of the PK sampling day according to the protocol (no major protocol deviations).

All protocol deviations that occur during the study will be considered prior to database lock for their severity/impact and will be taken into consideration when subjects are assigned to analysis populations. Details of subject assignment to the analysis populations will be listed.

All statistical analyses and summary of biomarker data will be performed on the PP population, whilst all summaries of the PK data will be performed on the PK population, and all summaries of the safety data will be performed on the safety population.

1. Statistical Methodology
   1. General

Data listings will be provided for the Safety Population. Summary statistics and statistical analyses will be performed for subjects included in the relevant analysis populations (Safety/ITT/PP/PK).

For continuous data, summary statistics will include the arithmetic mean, arithmetic standard deviation, median, minimum, maximum, and number. For log-normal data (e.g., the PK parameters: areas under the concentration-time curve [AUCs] and maximum observed concentration [C_max_]), the geometric mean, geometric coefficient of variation (CV%), and 95% confidence interval (CI) will also be presented. For the biomarker data, product use count and puff count during single use PK day the 95% CI will also be presented. For categorical data, frequency counts and percentages will be presented. Data listings will be provided for all subjects up to the point of withdrawal, with any subjects excluded from the relevant population highlighted. For the calculation of summary statistics and statistical analysis, unrounded data will be used.

Missing values will not be imputed.

Data analysis will be performed using SAS^®^ Version 9.3.

Analysis Data Model (ADaM) datasets will be prepared using Clinical Data Interchange Standards Consortium (CDISC) ADaM Version 2.1, and CDISC ADaM Implementation Guide Version 1.1. Pinnacle 21 Community Validator Version 2.1.2 will be utilised to ensure compliance with CDISC standards.

### Definition of Baseline and Change from Baseline

For the biomarker data the mean of the two values taken prior to first randomised product use (e.g. Day -1 to 1 and Day 1 to Day 2) will be used as the baseline value. If a value is missing then the other will be used as the baseline value. If a value is considered erroneous then the other value will be used as the baseline value. An erroneous value is defined as any outlier that can be explained. A discussion pre-lock of the biomarker data will be held to define any erroneous values.

For all other parameters, baseline for each parameter is defined as the last value measured prior to first randomised product use, including repeat (vital signs and ECGs) and unscheduled (clinical laboratory parameters) readings (see Section **Error! Reference source not found.** for definitions of repeat and unscheduled readings).

Mean change from baseline is the mean of all individual subjects’ change from baseline values. Each individual change from baseline will be calculated by subtracting the individual subject’s baseline value from the value at the timepoint. The individual subject’s change from baseline values will be used to calculate the mean change from baseline using a SAS procedure such as Proc Univariate.

Mean percent change from baseline is the mean of all individual subjects’ percent change from baseline values. Each percent change from baseline will be calculated by subtracting the individual subject’s baseline value from the value at the desired timepoint and then dividing this calculated value by the individual subject’s baseline value and multiplying by 100. These individual subjects’ percent changes from baseline values will be used to calculate the mean percent change from baseline using a SAS procedure such as Proc Univariate.

### Repeat and Unscheduled Readings

Repeat readings occur when the original vital signs or ECG result requires confirmation. Repeat readings are labelled as ‘Repeat’ in the listings and replace the original readings in all summaries and changes from baseline presentations and calculations. Prior to first randomised product use, all readings taken in addition to the original reading are defined as pre-first randomised product use repeats. Post-first randomised product use repeat readings are defined as readings collected within 15 minutes of the actual time of the original reading.

Unscheduled readings are labelled as ‘Unscheduled’ in the listings. Because unscheduled readings are not associated with any scheduled timepoint, they are excluded from all summaries (with the exception that they may be used as baseline, as stated in Section **Error! Reference source not found.**).

- 1. Demographics and Subject Disposition

The demographic variables age, sex, race, ethnicity, body weight, height, and body mass index will be summarised and listed. Subject disposition will be summarised and listed.

- - 1. Baseline Smoking Questionnaire

**Tobacco and Nicotine Use History Questionnaire**

The responses to all questions will be listed. The average number of cigarettes smoked per day, the brand of cigarette and the ISO tar rating will be summarised by randomised product group.

**Fagerström Test of Cigarette Dependence (FTCD)**

The FTCD total score will be derived by summing the individual question scores (see below) if all questions are non‑missing, otherwise the total score will be set to missing. The FTCD total score will be listed, and summarised, and frequency tables according to the following classification will also be provided for: Mild (0-3), Moderate (4-6), Severe (7-10). Individual question scores will be listed only.

| Scoring for the Fagerström Test for Cigarette Dependence | | | |
| --- | --- | --- | --- |
| **FTCD Question** | | **Response** | **Score** |
| 1 | How soon after you wake up do you smoke your first cigarette? | - Within 5 minutes - 6 to 30 minutes - 31 to 60 minutes - After 60 minutes | 3 2 1 0 |
| 2 | Do you find it difficult to refrain from smoking in places where it is forbidden? | - Yes - No | 1 0 |
| 3 | Which cigarette would you hate most to give up? | - The first one in the morning - Any other | 1 0 |
| 4 | How many cigarettes per day do you typically smoke? | - 10 or less (up to ½ pack) - 11 to 20 (a little more than ½ pack, up to a full pack) - 21 to 30 (a little more than a pack, up to 1½ packs) - 31 or more (more than 1½ packs) | 0  1  2  3 |
| 5 | Do you smoke more frequently during the first hours after waking than during the rest of the day? | - Yes - No | 1 0 |
| 6 | Do you still smoke if you are so sick that you are in bed most of the day? | - Yes - No | 1 0 |

- - 1. Medical History

Medical history data will be coded using the Medical Dictionary for Regulatory Activities (MedDRA) and listed.

- 1. Pharmacokinetic Assessment

### Pharmacokinetic Analysis

The following pharmacokinetic parameters will be determined where possible from the plasma concentrations of nicotine (arms A-E only) using non-compartmental methods, performed using Phoenix WinNonlin (Version 6.4 or higher):

| Parameter | Definition |
| --- | --- |
| AUC_0‑last_ | Area under the nicotine concentration-time curve from time 0 to the time of last quantifiable concentration (t_last_) |
| C_max_ | Maximum observed nicotine plasma concentration |
| t_max_ | Time of maximum observed nicotine plasma concentration |

Additional pharmacokinetic parameters may be determined where appropriate.

Pharmacokinetic analysis will, where possible, be carried out using actual postdose times recorded in the raw data. If actual times are missing, nominal times may be used with sponsor approval.

Concentrations are used as supplied by the analytical laboratory for PK analysis. The units of concentration and resulting PK parameters, with amount or concentration in the unit, will be presented as they are received from the analytical laboratory.

C_max_ and t_max_ will be obtained directly from the plasma concentration-time profiles. AUC_0‑last_ will be calculated using the linear trapezoidal rule for increasing concentrations and the logarithmic rule for decreasing concentrations.

For multiple peaks, the highest postdose concentration will be reported as C_max_. In the case that multiple peaks are of equal magnitude, the earliest t_max_ will be reported.

### Criteria for handling concentrations below the limit of quantification in Pharmacokinetic analysis

- Concentration values that are below the limit of quantification (BLQ) will be set to zero, with defined exceptions as follows;
  - Any embedded BLQ value (between 2 quantifiable concentrations) and BLQ values following the last quantifiable concentration in a profile will be set to missing for the purposes of PK analysis.
  - If there are late positive concentration values following 2 BLQ concentration values in the apparent terminal phase, these values will be evaluated. If these values are considered to be anomalous, they will be set to missing.
  - If an entire concentration-time profile is BLQ, the profile will be excluded from the PK analysis.
  - If a predose concentration is missing, these values may be set to zero.

### Calculation of AUC

- The minimum requirement for the calculation of AUC will be the inclusion of at least three consecutive plasma concentrations above the lower limit of quantification, with at least one of these concentrations following C_max_.
- For any partial AUC determination (i.e. AUC over a dosing interval), nominal time will generally be used for the end of the interval. Actual times for partial AUC intervals may be used at the discretion of the Pharmacokineticist.

### Anomalous Values

- If a value is considered to be anomalous due to being inconsistent with the expected pharmacokinetic profile, it may be appropriate to exclude this point from the pharmacokinetic analysis. However, the exclusion of data must have strong justification and will be documented in the raw data and study report.
- Embedded BLQ values may be considered anomalous depending on the route of administration and the characteristics of the drug.
  1. Presentation of Pharmacokinetic Data

### Presentation of Pharmacokinetic Plasma Drug Concentration Data

- The following rules will be applied if there are values that are BLQ or if there are missing values (e.g., no result [NR]) in a plasma concentration data series to be summarized.
  - For the calculation of summary statistics, BLQ values will be set to zero.
  - If an embedded BLQ value is considered anomalous within the concentration‑time profile, this value will be excluded from the summary statistics.
  - Where there is NR, these will be set to missing.
  - If there are less than three values in the data series, only the min, max and N will be presented. The other summary statistics will be denoted as not calculated (NC). BLQ is considered a value.
  - If all the values are BLQ, then the arithmetic mean, arithmetic SD, median, min and max will be presented as zero, and the geometric mean, geometric CV% and 95% CI will be denoted as NC.
  - If the value of the arithmetic mean or median is below the lower limit of quantification, these values will be presented as zero and the geometric mean, geometric CV% and 95% CI will be denoted as NC.

### Presentation Pharmacokinetic Parameters

- For the calculation of summary statistics of PK parameters, all NR and NC values in a data series will be set to missing.
- The AUC values will be set to NC if they have been calculated using fewer than three concentrations, and/or three concentrations if the last is C_max_.
  1. Biomarkers of Exposure

### Biomarker of Exposure Analysis Variables

The BoE on this study are:

- CO
- TNeq, where:

| TNeq [mg/24h] | = | (free nicotine[µmol/L] + nicotine-glucuronide [µmol/L]   + free cotinine [µmol/L] + cotinine-glucuronide [µmol/L]  + free trans-3’-hydroxycotinine [µmol/L]   + trans-3’-hydroxycotinine-glucuronide [µmol/L])  *162.2[µg/µmol]* (urine volume (L) / 1000) |
| --- | --- | --- |

N.B. All concentrations must be in µmol/L before applying the above formula. The sum of nicotine and 5 metabolite data will be listed. The conversion factors will be applied as follows:

| Free nicotine | The molecular weight is 162.232 g/mol. Therefore to convert nicotine from ng/mL to µmol/L, the result in ng/mL is multiplied by (6.164/1000). |
| --- | --- |
| Nicotine-glucuronide | The molecular weight is 338.356 g/mol. Therefore to convert nicotine‑glucuronide from ng/mL to µmol/L, the result in ng/mL is multiplied by (2.955/1000). |
| Cotinine | The molecular weight is 176.218 g/mol. Therefore to transform cotinine from ng/mL to µmol/L, the result in ng/mL will be multiplied by (5.675/1000). |
| Cotinine-glucuronide | The molecular weight is 352.341 g/mol. Therefore to transform cotinine-glucuronide from ng/mL to µmol/L, the result in ng/mL will be multiplied by (2.838/1000). |
| Trans-3’hydroxycotinine | The molecular weight is 192.217 g/mol. Therefore to transform trans-3’ hydroxycotinine from ng/mL to µmol/L, the result in ng/mL is multiplied by (5.202/1000). |
| Trans-3’hydroxycotinine-  glucuronide | The molecular weight is 368.34 g/mol. Therefore to transform trans-3’hydroxycotinine-glucuronide from ng/mL to µmol/L, the result in ng/mL is multiplied by (2.715/1000). |

- Total NNAL
- Total NNN
- 3-HPMA
- HMPMA
- S-PMA
- MHBMA
- CEMA
- 4-ABP
- o-Tol
- 2-AN
- 1-OHP
- HEMA
- AAMA and GAMA
- Nicotine metabolite ratio [3-hydroxycotinine (µmol/L) / cotinine (µmol/L)] using the conversion formulae given above. Calculated at baseline and Day 7 only.

For biomarkers of exposure in urine listed above (all apart from CO), the primary analysis variable is the amount excreted in 24 hours (Ae_24h_), defined as:

Ae24h [ng] = Urine concentration (ng/mL) * urine volume (mL).

N.B. If urine concentration is in pg/mL then convert to ng/mL by dividing the result in pg/mL by 1000. If the urine concentration is below the lower limit of detection or quantification, e.g. <20 ng/mL, then the urine concentration will be replaced by half the limit of detection or quantification, e.g. 10 ng/mL, prior to the calculation of the amount excreted. Similarly if the urine concentration is above the upper limit of quantification, e.g. >500 ng/mL, then the urine concentration will be replaced by the upper limit of quantification, e.g. 500 ng/mL, prior to the calculation of the amount excreted.

Please note that equation above for TNeq calculates the Ae_24h_ and no further calculation is required.

The table below shows the units to be used in the presentation of the amount excreted in the BoE.

| Biomarker | Units |
| --- | --- |
| TNeq | ng/24 h |
| Total NNAL | ng/24 h |
| Total NNN | ng/24 h |
| 3-HPMA | μg/24 h |
| HMPMA | μg/24 h |
| S-PMA | μg/24 h |
| MHBMA | ng/24 h |
| CEMA | μg/24 h |
| 4-ABP | ng/24 h |
| o-Tol | ng/24 h |
| 2-AN | ng/24 h |
| 1-OHP | ng/24 h |
| HEMA | μg/24 h |
| AAMA | μg/24 h |
| GAMA | μg/24 h |

### Biomarker of Exposure Statistical Methodology

The amount excreted for the urinary biomarkers over 24 h and the concentrations for CO in exhaled breath will be summarised along with actual changes and percentage changes from baseline at each measurement. The arithmetic mean actual and percentage changes from baseline will also be presented in a figure. The 24 h urine collection data, concentrations, the amount excreted, for each visit will be listed.

The baseline and Day 7 values will be used to investigate the within-arm changes in biomarkers for each arm separately using a paired t-test. Only subjects who have both baseline and Day 7 data will be included in the analysis. The SAS code will be similar to below:

proc ttest data=xxxx;

paired baseline*Day_7;

run;

The means will be reported for each day and arm separately. For each arm the mean difference between the Day 7 value and the baseline value will also be presented along with the 95% CI.

The baseline and Day 7 values will also be used to investigate comparisons between arms. A mixed ANOVA)^4^ with fixed terms for site, product use, day and arm and a random term for subject. The SAS code will be similar to below:

proc mixed data=xxxx;

class site day arm subject

model response= site day product_use arm day*arm;

random subject / subject=subject;

lsmeans day*arm / pdiff cl alpha=0.1;

ods output lsmeans=lsm;

ods output diffs=diff;

run;

where product use is defined as the number of cigarettes per day or sticks per day recorded at baseline and Day 7 by the subject. The baseline value corresponds to that used for the biomarker, i.e. the mean of the two days, or just the relevant day, as appropriate. If site is not significant (P>0.05) then do not include in the model.

The LS means difference from baseline will be reported for each product separately. For each comparison listed below the difference in the changes from baseline between the two products will also be presented along with the 95% CI.

The following comparisons (Test - Reference) will be performed:

- Arm B - Arm A
- Arm B - Arm F
- Arm D - Arm C
- Arm D - Arm F
- Arm A - Arm F
- Arm C - Arm F
- Arm E - Arm A

Prior to the analysis data will be checked for normality and may be transformed if considered appropriate. If transformed prior to the analysis the LS means and 95% CI will be back transformed to be presented on the original scale.

24-hour urine collection data, concentrations, the amount excreted, for each visit will be listed.

- 1. Biomarkers of Biological Effect

### Biomarker of Biological Effect Analysis Variables

The BoBE on this study are:

- 8-Epi-PGF2α Type III
- 8-Epi-PGF2α Type III adjusted for creatinine
- Total WBC count

The urinary creatinine adjusted excretion for 8-Epi-PGF2α Type III data will be calculated as (urine 8-Epi-PGF2α Type III concentration / urine creatinine concentration) x 1000.

### Biomarker of Biological Effect Statistical Methodology

The same outputs as described in section **Error! Reference source not found.** for the urinary biomarkers will be produced for the 8-Epi-PGF2α Type III data with, in addition, results adjusted for amount of creatinine. The same outputs as described in section **Error! Reference source not found.** for the CO concentrations will be produced for the WBC data.

The amount excreted of 8-Epi-PGF2α Type III will be presented in ng/24 h.

- 1. Safety and Tolerability Assessments

### Adverse Events

The AEs will be classed as occurring in one of three periods:

1. **Pre-enrolment** - any AE that starts after the subject has provided written informed consent and that resolves prior to enrolment on the study (Day -1), or an AE that starts prior to enrolment and does not increase in severity after enrolment.
2. **Baseline Period** - any AE that starts after the subject has enrolled on the study (Day -1) and that resolves prior to first use of a randomised product, or an AE that starts prior to first use of a randomised product and does not increase in severity after first use of a randomised product.
3. **Exposure Period** - any AE that occurs after the first use of the randomised product on Day 2 or that is present prior to the first use of the randomised product on Day 2 and becomes more severe after the first use of the randomised product on Day 2.

All AEs will be listed.

The AEs occurring in the baseline period will be summarised by arm the subject will be randomised to, severity, and relationship to the product. The frequency (the number of AEs, the number of subjects experiencing an AE, and the percentage of subjects experiencing an AE) of AEs will be summarised by arm the subject will be randomised to, and by MedDRA system organ class and preferred term. The summary and frequency AE tables will be presented for all causalities and for those AEs considered related to the product (those that have a relationship of possibly related or related). Any severe or serious AEs will be tabulated. For any AEs that change severity ratings the AE will be included only once under the maximum severity rating in the summaries.

The same set of summaries will be provided separately for the AEs occurring in the exposure period.

Onset times post product use are calculated from the last product administered.

### Clinical Laboratory Parameters

Clinical laboratory values will be listed and any values outside the clinical reference ranges will be flagged in the individual subject data listings.

### Vital Signs

Vital signs values will be listed and any values outside the clinical reference ranges will be flagged in the individual subject data listings.

### Electrocardiogram

Any clinically significant ECG interpretations will be listed.

### Lung Function Tests

The lung function test data will be recorded with and without salbutamol. These data include the Forced Vital Capacity (FVC), the Forced Expiratory Flow 25-75% (FEF_25-75%_), and the Forced Expiratory Volume after 1 second (FEV_1_). Predicted FEV_1_ and FVC will be calculated according to guidelines from the Japanese Respiratory Society^6^.

Lung function test values will be listed.

### Physical Examination

The date and time of all physical examinations will be listed along with any clinically significant results..

### Other Safety Assessments

All other safety assessments not detailed in this section will be listed but not summarised or statistically analysed.

- 1. Other Assessments

### Product Satisfaction

The questionnaire consists of 1 question, “Can you tell me how much do you like this tobacco product?”, which will be answered by the subject himself/herself. The subject will provide responses on a 7-point Likert scale ranging from “1 – I dislike it a lot” to “7 – I like it a lot” (scores of 2 – 6 will not have a descriptor).

These data will be listed and summarised.

### Product Use

The number of conventional cigarettes smoked or THP sticks used will be recorded over continuous 24-hour periods (concurrent with urine collection periods) from the evening of Day ‑1 until the beginning of the Exposure Period (Arm F) or the evening of Day 7 (Arms A to E).

These data will be listed and summarised. In addition a plot of the arithmetic mean (± SD) will be produced.

### Puff Count

The number of puffs taken by the subject on either a conventional cigarette or on the assigned THP product (Glo THP or iQOS) during the single product use in the PK Assessment Period (Day 8) will be listed and summarised. In addition a plot of the arithmetic mean (± SD) will be produced.

1. INTERIM analyses

An interim statistical analysis is planned after subjects using products A and B have completed the study. The following outputs will be sent for this interim analysis:

- Plots for each BoE separately showing amount excreted in 24h (or ppm for exhaled CO) for products A and B.
- A figure showing all BoE presenting the percentage change from baseline on Day 7 for products A and B.
- Summary table of percentage change from baseline on Day 7 for each BoE for products A and B.
- A paired t-test comparing the baseline and Day 7 values for the amount excreted (or ppm for exhaled CO) for each BoE separately for products A and B.

1. changes from the protocol specified statistical analyses

The definition of the PK population has been slightly amended prior to unblinding to state that subjects only need to complete up to the end of the PK sampling day rather than the whole study, so that we do not exclude anybody from the population who only do not complete the follow-up period.

1. DAta Presentation
   1. Insufficient Data for Presentation

Some of the TFLs may not have sufficient numbers of subjects or data for presentation. If this occurs, the blank TFL shell will be presented with a message printed in the center of the table, such as, “No serious adverse events occurred for this study.”

1. References
2. International Conference on Harmonisation of Technical Requirements for Registration of Pharmaceuticals for Human Use, ICH Harmonised Tripartite Guideline, Statistical Principles for Clinical Trials (E9), 5 February 1998.
3. International Conference on Harmonisation of Technical Requirements for Registration of Pharmaceuticals for Human Use, ICH Harmonised Tripartite Guideline, Structure and Content of Clinical Study Reports (E3), 30 November 1995.
4. Snedecor GW, Cochran WG. Statistical Methods (8th edition). Iowa: Iowa State Univ Press, 1982: 217-253.
5. Lehmann EL. Nonparametrics: Statistical Methods Based on Ranks. New York: McGraw‑Hill, 1975; Ch 1.
6. Brown H, Prescott R. Applied Mixed Models in Medicine. Wiley, 1999; Chs 1 & 2.
7. Japan Respiratory Society. Respiratory function test guideline – spirometry, flow volume curve, pulmonary diffusion capacity. 2004. Tokyo: Medical Review Co., Ltd.
